# Promising Antibacterial and Antifungal Agents Based on Thiolated Vitamin K3 Analogs: Synthesis, Bioevaluation, Molecular Docking

**DOI:** 10.3390/ph15050586

**Published:** 2022-05-10

**Authors:** Hatice Yıldırım, Mahmut Yıldız, Nilüfer Bayrak, Emel Mataracı-Kara, Mohamed Osman Radwan, Ayse Tarbin Jannuzzi, Masami Otsuka, Mikako Fujita, Amaç Fatih TuYuN

**Affiliations:** 1 Department of Chemistry, Faculty of Engineering, Istanbul University-Cerrahpasa, Avcilar, Istanbul 34320, Turkey; hyildirim@istanbul.edu.tr (H.Y.); nbayrak@istanbul.edu.tr (N.B.); 2 Department of Chemistry, Gebze Technical University, Gebze, Kocaeli 41400, Turkey; yildizm@gtu.edu.tr; 3 Department of Pharmaceutical Microbiology, Faculty of Pharmacy, Istanbul University, Beyazit, Istanbul 34116, Turkey; emataraci@istanbul.edu.tr; 4 Medicinal and Biological Chemistry Science Farm Joint Research Laboratory, Faculty of Life Sciences, Kumamoto University, 5-1 Oe-honmachi, Chuo-ku, Kumamoto 862-0973, Japan; mohamedosman251@gmail.com (M.O.R.); motsuka@gpo.kumamoto-u.ac.jp (M.O.); mfujita@kumamoto-u.ac.jp (M.F.); 5 Chemistry of Natural Compounds Department, Pharmaceutical and Drug Industries Research Division, National Research Centre, Dokki, Cairo 12622, Egypt; 6 Department of Pharmaceutical Toxicology, Faculty of Pharmacy, Istanbul University, Beyazit, Istanbul 34116, Turkey; tarbin.cevik@instanbul.edu.tr; 7 Department of Drug Discovery, Science Farm Ltd., 1-7-30 Kuhonji, Chuo-ku, Kumamoto 862-0976, Japan; 8 Department of Chemistry, Faculty of Science, Istanbul University, Fatih, Istanbul 34126, Turkey

**Keywords:** antibacterial activity, antibiofilm activity, Vitamin K, *Staphylococcus aureus*, thymidylate kinase

## Abstract

In the present study, we designed and synthesized thiolated **VK3** analogs (**VK3a–g**) along with an extensive antimicrobial study. After the evaluation of the antibacterial and antifungal activity against various bacterial and fungal strains, we presented an initial structure–activity relationship study on these **VK3** analogs. In particular, four thiolated **VK3** analogs exhibited superior biological potency against some Gram-positive bacterial strains, including *Staphylococcus aureus* (ATCC^®^ 29213) and *Enterococcus faecalis* (ATCC^®^ 29212). Next, all thiolated **VK3** analogs were evaluated for their potential of cell growth inhibition on the NCI-60 cancer cell lines panel. This screening underlined that the thiolated **VK3** analogs have no visible cytotoxicity on different cancer cell lines. The selected two thiolated **VK3** analogs (**VK3a** and **VK3b**), having minimal hemolytic activity, which also have the lowest MIC values on *S. aureus* and *E. faecalis*, were further evaluated for their inhibition capacities on biofilm formation after evaluating their potential in vitro antimicrobial activity against each of the 20 clinically obtained resistant strains of *Staphylococcus aureus*. **VK3b** showed excellent antimicrobial activity against clinically resistant *S. aureus* isolates. Furthermore, the tested molecules showed nearly two log_10_ reduction in the viable cell count at six hours according to the time kill curve studies. Although these molecules decreased biofilm attachment about 50%, when sub-MIC concentrations were used these molecules increased the percentage of biofilm formation. The molecular docking of **VK3a** and **VK3b** in *S. aureus* thymidylate kinase was conducted in order to predict their molecular interactions. **VK3a** and **VK3b** exhibited excellent lead-likeness properties and pharmacokinetic profiles that qualify them for further optimization and development. In conclusion, since investigating efficient novel antimicrobial molecules is quite difficult, these studies are of high importance, especially in the present era of antimicrobial resistance.

## 1. Introduction

**Figure 1:**
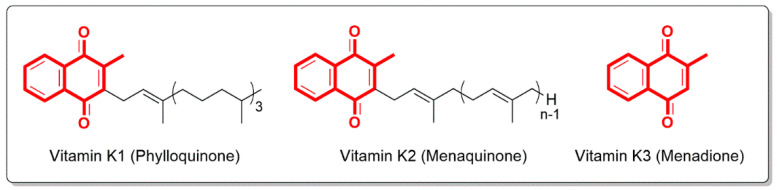
The most important members of Vitamin K family.

Vitamins are micronutrients comprised of organic structures that cannot be synthesized by the human body, but must be taken exogenously for the body in order to perform its normal functions and prevent metabolic disorders [1]. It is known that a diet rich in vitamins strengthens the immune system and plays an important role in fighting against disease-causing microorganisms. Additionally, although data on the protective factors of the disease are limited in the Coronavirus epidemic, which has turned into a worldwide pandemic, vitamins are among the preventive health measures that can reduce the risk of infection in elderly and immunocompromised individuals [2,3]. The most significant vitamins for the human body are vitamin A [4], vitamin B complex [5], vitamin C [6], vitamin D [7], and Vitamin K [8] etc. Vitamin K is an important component in the system playing a key role in blood coagulation in addition to strengthening bones and promoting the calcification of arteries and soft tissues [9]. The structure of the Vitamin K family consists of a common motif structure 2-methyl-1,4-naphthoquinone analogs, as shown in Figure 1. Vitamin K3 (**VK3**), named as menadione, is a synthetic analog of natural Vitamin K, unlike the other two forms, which exist in nature (Figure 1) [10,11]. **VK3** analogs are attractive to medicinal and/or organic chemists as a result of their unique chemical and broad range of biological activities [12,13]. Such research indicates that the **VK3** moiety is an important framework with good biological potency, which effectively shows anticancer [14,15], antimalarial [16,17], antioxidant [18], and catalase inhibition activity [19,20]. In addition to inhibition of in vitro and in vivo cancer cell lines [21,22,23,24,25], iron-**VK3** analog was shown to synergistically promote ferroptotic therapy for inhibiting tumor growth, preventing metastasis and tackling radioresistance [26].

**Figure 2:**
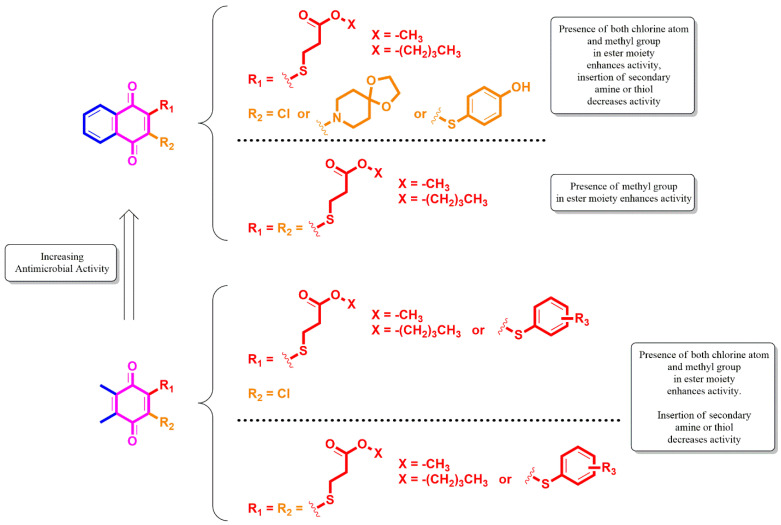
Design strategy by the incorporation of **VK3** and alkyl chain thiols as the substrates, based on previous results from the literature.

For some time in our laboratory, we have focused on the construction of 1,4-quinones with different substitution patterns, along with detailed studies on their potential biological performances, in order to discover new compounds that have the potential to be lead molecules in the fight against cancer and antimicrobial resistance [27,28,29]. To date, the biologically important lead structures have been obtained and gathered for the tuning of pharmacological efficacy as an antimicrobial and/or anticancer agent. It is mainly understood that incorporating amino groups (i.e., aryl amines, piperazines, and piperidines) into 1,4-quinone moiety with different side groups (i.e., phenyl, dimethyl, and pyridinyl) has mainly increased the biological potency of the target molecules [27,28,29]. On the other hand, we have previously accessed the C2- and/or C2C3-thiolated 1,4-quinones with different side groups, such as the dimethyl or pyridinyl group [30,31]. A key aspect of the biological activity in our previous studies is the certain chemical modifications of 1,4-quinone moiety. Naphthoquinones [19,32,33,34,35], quinolinequinones [27,30,36,37], and dimethylbenzoquinones [28,38,39] are the most widely used motifs belonging to the quinone family, being biologically important lead structures, which have been extensively used over the years [40,41]. Some of these C2- and/or C2C3-thiolated 1,4-quinones with different side groups were more potent than the reference drug(s). In these studies, alkyl chain thiols were connected to a dichloronaphthoquinone [32,33] and dimethylbenzoquinone [28,38,39,42] via different substrates [30,31]. As seen in these studies, the used alkyl chain or aromatic thiols as substrates, connected to dimethylbenzoquinone, did not exhibit any significant antibacterial and/or antifungal activity. Last but not least, the introduction of the dichloronaphthoquinone in alkyl chain thiols gave the powerful lead structures for antimicrobial (antibacterial and antifungal) studies [32,33]. Another well-explored moiety of dichloronaphthoquinone is the second halogen atom, which easily undergoes substitution reactions with additional amines or thiols. Replacement of the chlorine atom with either amines or thiols decreased the antimicrobial potency. Some works were focused on the synthesis of these mixed thiolated naphthoquinones and aminated thiolated naphthoquinones (Figure 2) [32,33,43,44,45,46]. In general, the presence of the alkyl chain thiol and the chlorine atom is more important for the activity than fully thiolated naphthoquinones [32,33]. Thus, to reveal the effect of the quinone moiety and the second substituent (chloro, amino, or thio group) on the antimicrobial potency, **VK3** (Menadione) was selected as a parent molecule of the substrates to synthesize some analogs of **VK3**. Motivated by these studies and given our long-standing interest in these specific structures, we envisioned that the used alkyl chain thiols as the substrates might offer new lead structures against antimicrobial resistance. In this paper, efforts for the design, synthesis, and antimicrobial evaluation of target analogs of **VK3** were made to exploit the effect of structural modification on their potency obtained from the commercially available **VK3** under mild conditions. Indeed, some members of thiolated **VK3** were investigated for their antibiofilm activity, along with their potential antimicrobial activity against each of the 20 clinically obtained strains of Methicillin resistant *Staphylococcus aureus*, and bactericidal time-kill kinetic study.

## 2. Results and Discussion

### 2.1. Library Design and Synthesis

**Scheme 1:**
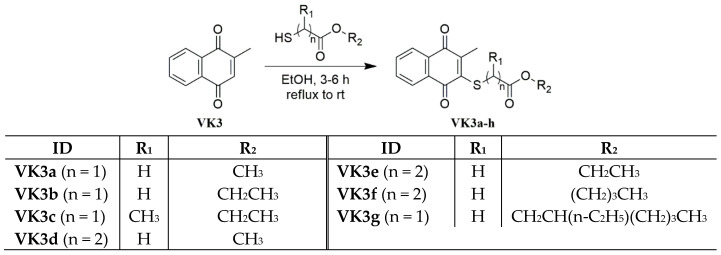
Construction of the thiolated **VK3** analogs (**VK3a**–**g**).

**Figure 3:**
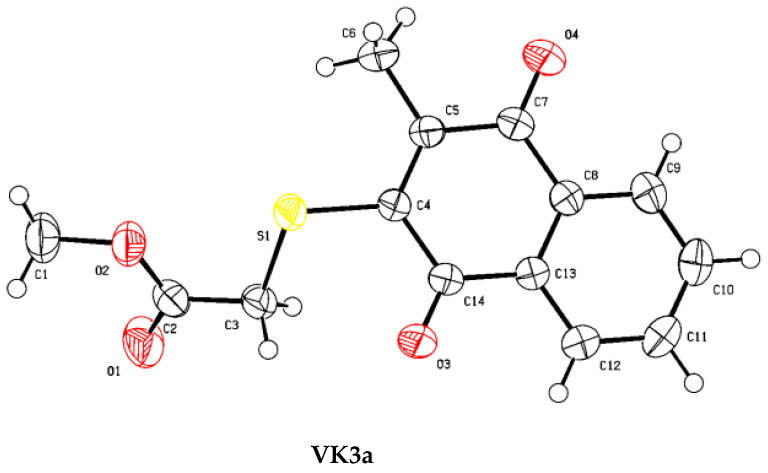
ORTEP drawings of **VK3a** at 50% probability level.

Due to their high electrophilicity, versatile 1,4-quinones are very reactive towards thiols, which are soft nucleophiles according to the Pearson’s scale, unlike primary and secondary amines. Our synthesis studies suggest that **VK3** has only one capacity to react with alkyl chain thiols under mild condition. The synthetic strategy adopted for the synthesis of the target **VK3** analogs (**VK3a–g**) is depicted in one step (Scheme 1). The commercially available **VK3** was directly thiolated using the corresponding alkyl chain thiols to give the target **VK3** analogs (**VK3a–g**) in ethanol in one step (Scheme 1) by adopting the procedure with a slight modification (i.e., heating) from that mentioned in the literature [47]. The reaction mixture was then refluxed for three–six hours to yield the final desired **VK3** analogs. (**VK3a–g**) were purified via silica gel column chromatography. All of the thiolated **VK3** analogs were fully characterized by mass spectroscopy (MS), Fourier-transform infrared spectroscopy (FTIR), nuclear magnetic resonance (NMR), and high-resolution mass spectrometry (HRMS). The structure of methyl 2-(3-methyl-1,4-dioxo-1,4-dihydronaphthalen-2-ylthio)acetate (**VK3a**) was determined by single-crystal X-ray diffraction analysis (Figure 3). The absorption bands from vibrations of the following bands are observed at around 1720 and 1650 cm^−1^ for C=O; at around 1590 cm^−1^ for C=C; 2900 cm^−1^ for C-H_aliphatic_ in the IR spectra. The methylene protons attached to either the oxygen or sulfur atom were observed at around 4.00 ppm. In some cases, the methylene protons attached to the sulfur atom were seen at around 3.50 ppm. The singlet in the region of 3.70 ppm was assigned to the protons of the methyl group attached to the oxygen atom, and multiplets or doublets in the region of 6.90–8.00 ppm to the protons of aromatic rings. The ^13^C NMR shifts of the carbonyl carbon atoms, within 1,4-quinone of the target analogs, appeared downfield at around 182 and 181 ppm as two peaks, while the other carbonyl carbon atoms, which belong to thiol moiety, had resonance at around 170 ppm. The individual -OCH_2_ and -OCH_3_ carbon atoms in each molecule of the library provided chemical shift values of around 63 ppm and 53 ppm, respectively. The methyl carbon atoms connected to the 1,4-quinone moiety have resonanced at upfield around 14 ppm. Additionally, the methylene carbons attached to the sulfur atom within thiol moiety were observed at around 40 ppm. Moreover, in the high-resolution mass spectra of the thiolated **VK3** analogs, the molecular ion peak with a proton adduct were detected in strong abundance.

To produce **VK3a** in high purity, the compound was dissolved in ethanol and stored for at least one week for crystallization. The structure of **VK3a** was analyzed by the X-ray diffractometer. The ORTEP drawing (displacement parameters drawn at 50% probability level) of the **VK3a** is shown in Figure 3. The crystal system of **VK3a** is monoclinic (space group P 1 21/n 1). It is clear from the X-ray analysis data that the bond lengths of O1-C2 and O2-C2 belonging to the ester group of **VK3a** are 1.19 Å and 1.32 Å, respectively (Table 1). In the data of X-ray analysis, the bond lengths of O1-C2 and O2-C2 of the ester group of **VK3a** are 1.19 Å and 1.32 Å, respectively. It is clear that the bond between O2 and C2 atoms is longer than the bond between C2 and O1 atoms, and that the O1-C2 bond has a characteristic double bond and the O2-C2 bond has a characteristic single bond. By experimentally observing that the torsion angle of S1-C4-C14-O3 is 5.4°, it can be determined that the sulfur and oxygen atoms are approximately in the same plane. From the determination of the torsional angle of C3-S1-C4-C14 as −49.5°, it can be deduced that the methylene group may have intramolecular and intermolecular H-bonding interaction with the carbonyl group. The X-ray data of **VK3a** also shows H-bonding between the oxygen atom as an acceptor and methylene hydrogens as donors. The selected bond lengths, bond angles, torsion angles, hydrogen bond distances, and angles are given in Table 2, Table 3, Table 4 and Table 5.

### 2.2. Biological Activity

#### 2.2.1. Evaluation of Antimicrobial Activity of the Thiolated VK3 Analogs (**VK3a–g**)

The obtained thiolated **VK3** analogs (**VK3a–g**) were initially tested in vitro for their antibacterial and antifungal profile by determining their minimum inhibitory concentration (MIC) values, listed in Table 6 and Table 7 and compared with the commercially available reference drugs, against four Gram-negative bacteria (*Pseudomonas aeruginosa* ATCC 27853, *Escherichia coli* ATCC 25922, *Klebsiella pneumoniae* ATCC 4352, and *Proteus mirabilis* ATCC 14153), three Gram-positive bacteria (*Staphylococcus aureus* ATCC 29213, *Staphylococcus epidermidis* ATCC 12228, and *Enterococcus faecalis* ATCC 29212), and three fungi (*Candida albicans* ATCC 10231, *Candida* *parapsilosis* ATCC 22019, and *Candida* *tropicalis* ATCC 750) by the broth microdilutions technique using the Clinical Laboratory Standards Institute (CLSI) recommendations [48,49]. The MIC values were determined by comparison with standard agents.

According to the obtained results, the thiolated **VK3** analogs (**VK3a–g**) did not have any antibacterial activity against Gram-negative bacterial strains (*P. aeruginosa*, *E. coli*, *K. pneumoniae*, and *P. mirabilis*) in addition to one Gram-positive bacterial strain (*S. epidermidis*). It is noteworthy to mention that three of the thiolated **VK3** analogs (**VK3a-b** and **VK3d**) were the most active analogs on *S. aureus* and *E. faecalis* with MIC values of 4.88 and 39.06 μg/mL, respectively. Furthermore, two of them (**VK3c** and **VK3f**) were the other remarkable **VK3** analogs against *S. aureus* with MIC of 9.76 μg/mL. **VK3c** was another remarkable **VK3** analog on *E. faecalis*. When the obtained thiolated **VK3** analogs (**VK3a–g**) are evaluated in terms of the antifungal activity, **VK3b** showed high antifungal activity against *C. albicans* possessing the MIC value of 9.76 μg/mL. Besides, **VK3a** and **VK3d** were observed to be the most active **VK3** analogs as antifungal agents against *C. tropicalis* with 9.76 μg/mL MIC value. Among the tested **VK3** analogs, two **VK3** analogs (**VK3a** and **VK3b**) showed strong antibacterial and antifungal potency. Moreover, the antibacterial activity of these two analogs seems to be strongly specific to *S. aureus* and *E. faecalis* since their MIC values against other tested bacterial strains were relatively better. Thus, we mainly focused on these two **VK3** analogs for the best antimicrobial potency against clinically obtained Methicillin-resistant strains of *Staphylococcus aureus*. The in vitro activities of the **VK3a** and **VK3b** analogs against 20 clinical isolates of *MRSA* are summarized in Table 8. Although the in vitro activity of **VK3a** was found to have a moderate effect on the studied clinical strains, **VK3b** showed excellent activity on these isolates, similar to be the standard *S. aureus* ATCC isolate’s MIC results. Moreover, there was no major difference between the bactericidal and inhibitory endpoints; the MBCs were generally two-fold higher than those of the MICs. With these excellent activity results, especially from **VK3b**, we further analyzed the anticancer property of these antimicrobials.

Although the biological dataset is small, some structure–activity relationships can be extracted. Herein, to examine how the alkyl chain thiols affect the antibacterial and antifungal activity, we have varied the backbone group attached to the oxygen atom, while keeping the methylene bridge(s) or methine bridge between the sulfur and carbonyl group of the substrates. Keeping the methylene bridge unchanged, the group attached to the oxygen atom differed from the methyl or ethyl group. In this case, the activity was not affected. The activity against *S. aureus* was decreased with the insertion of an additional methyl group instead of a hydrogen atom in the methylene group between the sulfur atom and carbonyl group in the thiolated **VK3** analog (**VK3c**). When the methylene bridge was held with one additional methylene group, the analogs were diversified with alkyl groups such as methyl, ethyl, or butyl group. Thus, only one methylene bridge between the sulfur and carbonyl group are allowed in the analogs, with a methyl or ethyl group favoring inhibition rather than molecules having two methylene bridges between the sulfur and carbonyl group (**VK3a** vs **VK3d**; **VK3b** vs. **VK3e**). Replacement of a hydrogen atom with methyl group in the methylene bridge between the sulfur and carbonyl group decreased activity slightly (**VK3b** vs **VK3c**) against *S. aureus*, but had no effect against *E. faecalis*. In general, we could conclude that a remarkable decrease in inhibitory activity was observed when the length of the alkyl chain attached to the oxygen atom was changed from methyl to ethyl or butyl group. Upon increasing the alkyl group attached to the oxygen atom to varied groups, there was a remarkable decrease in activity against all the tested bacteria. In other words, bulkier substituents, such as long-chained alkyl groups in **VK3g,** are detrimental to the activity. Both the introduction of the second methylene bridge between the sulfur and carbonyl group and the length of the alkyl chain attached to the oxygen atom diminished the activity. The detrimental effect is stronger at the long alkyl chain attached to the oxygen atom than the methylene bridge. The same parabolic pattern was followed for the thiolated **VK3** analogs for antifungal activity.

#### 2.2.2. Toxicity Evaluation

In order to check the cell toxicity of the tested thiolated **VK3** analogs presented herein, all thiolated **VK3** analogs were submitted to the National Cancer Institute (NCI) of Bethesda within the Developmental Therapeutics Program (DTP). After selection, they were screened for evaluation of their cell growth inhibition potential on the NCI-60 cancer cell lines panel [50] at a single high dose concentration (10 µM) on nine different cancer types, namely: leukemia; lung; colon; CNS; melanoma; ovarian; renal; prostate; and breast cancer cell lines [51] with the protocol of the Drug Evaluation Branch, NCI [52]. Herein, testing of those analogs (**VK3a–g**) against the NCI panel of 60 cell lines provides us with comprehensive information on the growth inhibitory effects of analogs. The single-dose results, released in the form of a mean graph of the cell growth percentage (GP) of each of the tested thiolated **VK3** analogs, were reported. Their one-dose mean graphs are presented in the Supplementary Material as Figures S3–S9. The thiolated **VK3** analogs displayed slight, to completely no cytotoxic effects towards all of the cancer cell lines.

#### 2.2.3. Hemolytic Activity

**Figure 4:**
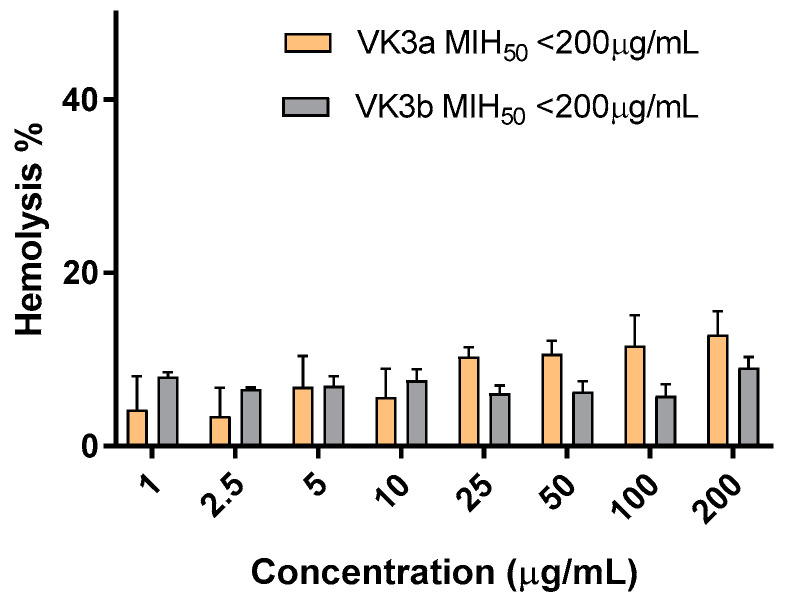
Hemolytic activity (% of hemolysis) of **VK3a** and **VK3b** in human erythrocytes within the concentration range of 1–200 µg/mL. (n = 3, MIH_50_: The minimal concentrations inducing 50% hemolysis).

The toxicity of **VK3a** and **VK3b** on erythrocytes was assayed with hemolysis assay. Even at the highest concentrations, which were 70 fold higher than the dose for the toxicity evaluation with NCI-60 cancer cell lines panel, both of the analogs had minimal hemolytic activity and the minimal concentrations inducing 50% hemolysis (MIH_50_) were <200 µg/mL for **VK3a** and **VK3b** (Figure 4). These results indicate that both **VK3a** and **VK3b** analogs showed a good safety profile and were proved to be devoid of hemolytic activity in a broad range of concentrations, which also covered their antimicrobial concentrations.

#### 2.2.4. Time-Kill Kinetic Study

**Figure 5:**
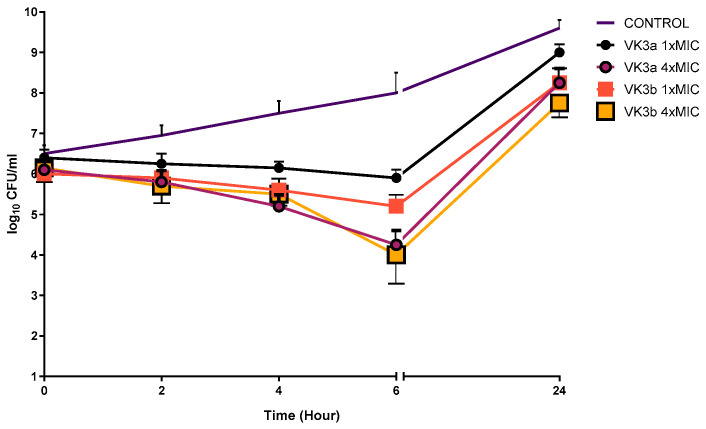
Time-kill determinations for clinically resistant *MRSA* isolate after treatment with **VK3a** and **VK3b** at 1× and 4× MIC, respectively. The *x*-axis represents the killing time, and the *y*-axis represents the logarithmic *MRSA* survival.

Due to their significant in vitro antimicrobial activity results, **VK3a** and **VK3b** were chosen between the tested molecules for further investigation of the mode of action. Time-kill studies for **VK3a** and **VK3b** were studied on one clinically obtained Methicillin resistant *Staphylococcus aureus* isolates and the results are given in Figure 5.

According to our Time-kill curve results, **VK3a** and **VK3b** did not show bactericidal activity (with a 3-log_10_ kill determined) against the studied strain at 1× and 4× MIC concentrations within 24 h. (Figure 5). Even if the tested molecules decreased the viable cell count at six hours for the tested strain, within 24 h, bacterial regrowth was seen when the tested molecules were used alone. Apart from our study, Ravichandiran et al. [44,45] showed that newly synthesized 1,4-naphthoquinone molecules had excellent bactericidal activity in a time-dependent manner at 24 h on the studied *Escherichia coli* and *Staphylococcus aureus* strains by using the time-kill curve method. The differences between the reduction in the bacterial cell count could be associated with the resistance profile of the tested microorganisms. Due to the promising antimicrobial activity especially on the clinically resistant isolates, even if bacterial regrowth was seen at 24 h in the time-kill curve studies, these molecules provide a chance to try the synergistic activity in combination with antibiotics frequently used in clinics to inhibit bacterial regrowth in studied strains in further investigations.

#### 2.2.5. Evaluation of the In Vitro Antibiofilm Activity

The occurrence of many biofilm-based infections and their multiple antimicrobial tolerance are major concerns in healthcare. The elevated rate of resistance to antibiotics in biofilm leads to the discovery of novel anti-biofilm agents. Biofilm formation mainly occurs in four stages: (1) bacterial attachment to a surface; (2) microcolony formation; (3) biofilm maturation; and (4) detachment (also termed dispersal) of bacteria, which may then colonize new areas [53,54]. Due to the increased antimicrobial tolerance with the matured biofilm, it is easier to target the elimination of the biofilm formation at the first two stages.

**Figure 6:**
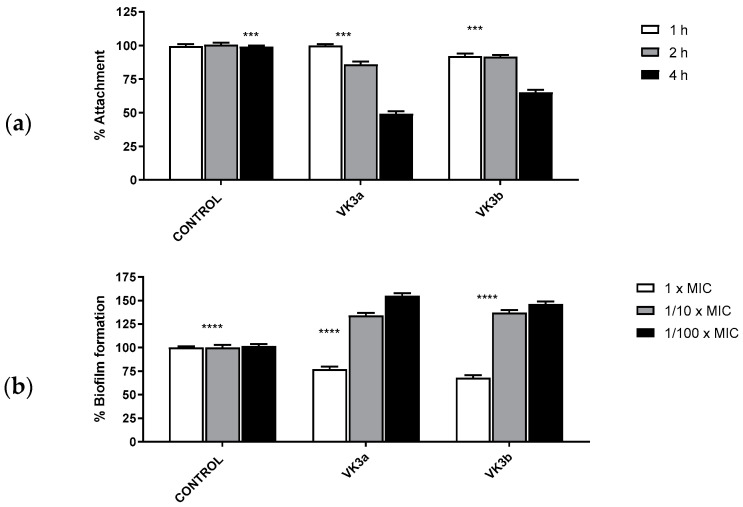
Inhibition of *MRSA*: (**a**) surface attachment to the wells contained 1/10× MIC of molecules and an inoculum of 1 × 10^6^ CFU/200 μL, incubated for 1, 2, or 4 h at 37 °C for *MRSA*; (**b**) biofilm formation in each well contained 1×, 1/10×, or 1/100× MIC of molecules and an inoculum of 1 × 10^6^–1 × 10^7^ CFU/200 μL, incubated for 24 h at 37 °C for *MRSA*. Control bars accepted as 100% indicate microorganisms without molecules. Six wells were used for the tested molecule. Each experiment is representative of two independent tests. All differences between the control and molecule treated biofilms were statistically significant (**** *p* < 0.005; *** not significant).

For this reason, we purposed to determine the antibiofilm effects of **VK3a** and **VK3b** against the first two stages of clinically obtained *MRSA* biofilms. When the 1/10× MICs of tested molecules were examined for 1, 2, or 4 h at 37 °C for *MRSA*’s adherence to the wells of tissue culture microtiter plates, the tested agents inhibited biofilm attachment processes at least 50% for **VK3a** at four hours. In general, the inhibition rates of adhesion showed a time-dependent effect for *MRSA* (Figure 6). When we evaluated the biofilm formation percentage of the studied strains, the rates of biofilm formation inhibition were dependent on concentration, and the highest inhibition rates were shown at 1× MICs for the tested molecules (Figure 6). However, when 1/10× MIC and 1/100× MIC concentrations were used, these molecules acted like substrates for bacterial growth. From the antibiofilm activity results, even if these molecules could achieve an almost 50% decrease in the biofilm attachment process after 4 h for the tested concentrations, at 1/10× MIC and 1/100× MIC (concentrations), these molecules seemed to increase biofilm formation. Our results, similar to Silva et al. [55] and Novais et al. [56] showed time and dose dependent antibiofilm activity against tested isolates.

#### 2.2.6. Molecular Docking Study

**Figure 7:**
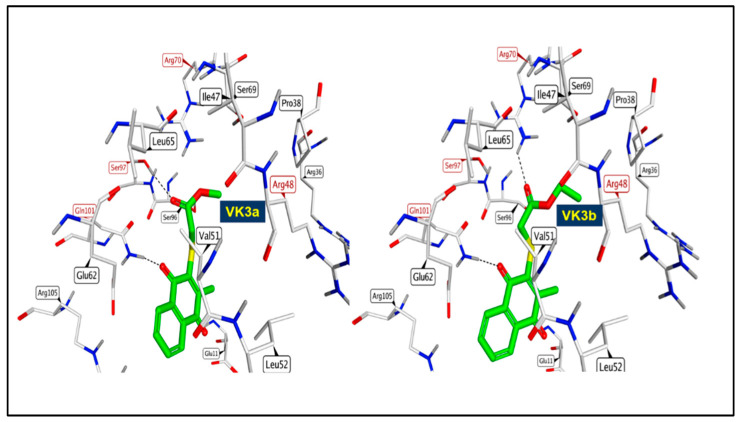
Binding mode of compounds **VK3a** (**left**) and **VK3b** (**right**) into *S. aureus* TMK (PDB:4GFD) showing similarity in their placement and interactions with the key amino acids.

Thymidylate kinase (TMK) is an enzyme that delivers phosphate from ATP to thymidine monophosphate to produce thymidine diphosphate [57,58]. Inhibition of bacterial TMK function blocks DNA biosynthesis and consequently leads to cell death [59]. To get more insights into the mechanism of action of **VK3a** and **VK3b**, we docked them into *S. aureus* thymidylate kinase (TMK), a nucleotide kinase for a bacterial DNA synthesis pathway and a valid target for the development of new antibacterial drugs [60,61,62]. The thymidine monophosphate site is deep and shares fewer common residues with human TMK, which makes it more druggable than the ATP binding site. Visualization of docked conformations of **VK3a** and **VK3b** into TMK from *S. aureus* showed that they are likely to adopt the same orientation and interactions. Side chain C=O group of **VK3a** and **VK3b** are involved in H bonding with key amino acids Ser97 and Arg70, respectively, whereas their C=O of the 1,4 quinone scaffold forms H bonds with Gln101 [63] (Figure 7). However, both compounds lack crucial interactions with Arg48. It seems that the extension of the side chain by using longer R_2_ leads to an atomic clash with the pocket amino acids, which explains the low activity of **VK3g** and **VK3h**.

#### 2.2.7. In Silico Drug-Likeness and ADME Analysis of Selected Thiolated VK3 Analogs

Drug-likeness prediction is a quantitative concept used to study the probability that a chemical compound could be a potential drug. SwissADME server, a free tool for drug-likeness prediction, was employed for calculating several molecular and structural features of **VK3a** and **VK3b**. Both compounds can be easily transported in the body due to their low molecular weight. This small molecular weight makes them ideal lead compounds that can be modified to enhance their affinity within the target enzyme. Their octanol-water partition coefficient (log P), which indicates lipophilicity, is in the acceptable range (−0.4–5.6) (Table 9). Their total polar surface area TPSA is less than 160 Å^2^ indicating good bioavailability. Furthermore, the number of hydrogen bond acceptors (HBA < 10) and the number of hydrogen bond donors (HBD < 5), are in the acceptable ranges (Table 9). SwissADME calculations revealed that the titled compounds have leadlikeness properties [64] and they obey all of Lipinski [65], Ghose [66], Veber [67], Egan [68], and Muegge [69] rules for druglikeness without any violation.

The ADME properties of **VK3a** and **VK3b** displayed promising profiles as well. In SwissADME, they use a BOILED-Egg model that showed that both compounds cannot pass through the blood brain barrier (BBB), which helps avoid detrimental effects on the central nervous system (CNS). They are also likely to have a good gastrointestinal (GI) absorption, which is attributed to their ability to be passively absorbed by the GIT. They have quite good solubility and bioavailability scores as shown in Table 9. In addition, they may interfere with the function of the metabolic enzymes cytochrome P450 isoforms CYP1A2, CYP2C19, and CYP2C9 without having an effect on the other isozymes CYP2D6 and CYP3A4.

## 3. Materials and Methods

### 3.1. Chemicals and Apparatus

All the solvents and reagents used in the present study were purchased from commercial suppliers with a high purity standard, and used without any purification prior to use. Thin-layer chromatography (TLC) was carried out with silica gel coated aluminum sheets purchased from Merck KGaA. Visualization was achieved using UV light (254 nm). All molecules were purified by column chromatography with Silica gel 60 (63–200 µm particle-sized). Melting points (mp) were measured in a capillary tube in an electrical melting point (Büchi B-540) and are uncorrected. Purified analogs were characterized by nuclear magnetic resonance spectroscopy (^1^H—500 MHz, ^13^C—125 MHz). The ^1^H NMR spectra data is expressed in the form: chemical shifts in units of parts per million (ppm) in CDCl_3_ and coupling constants (J) are in hertz (Hz). The infrared (IR) spectra of all analogs were obtained on a FTIR spectrometer, using the single reflection diamond ATR module. Mass spectra were performed on a BRUKER Microflex LT equipped with a MALDI (Matrix Assisted Laser Desorption Ionization)-TOF technique by the addition of 1,8,9-anthracenetriol (DIT, dithranol) as a matrix. High-resolution mass spectra electrospray ionization (HRMS-ESI) was obtained on a Waters SYNAPT G1 MS by dissolving analogs (2–3 mg) in acetonitrile. Prior to biological activity, the purity of the thiolated **VK3** analogs (**VK3a–g**) was confirmed by HPLC with hexane/2-propanol = 95:5 as the mobile phase at a flow rate of 1.0 mL/min. Purity of all thiolated **VK3** analogs (**VK3a–g**) was further confirmed to be ≥95% by HPLC analyses with Shimadzu/DGU-20A5 HPLC apparatus fitted with a 25 cm Chiralpac AD-H chiral column. Their chromatograms are provided as Supplementary Material (Figures S8–S14).

### 3.2. X-ray Diffraction Analysis

A Bruker APEX II QUAZAR three-circle diffractometer was used to obtain the data for the single crystal compounds. APEX2 was used to perform indexing [70]. SAINT was used for data integration and reduction [71]. Absorption corrections were performed by the multi-scan method implemented in SADABS [72]. The structure was solved by direct methods using the Bruker SHELXTL [73] software package. Structure refinement: full matrix least-squares methods on F2 using SHELXTL all non-hydrogen atoms with anisotropic displacement parameters. Platon software was used to validate the crystal structure and to calculate [74]. The .cif files were processed with Mercury software [75] for the visualization. Table 1 shows the crystallographic and structure refinement data in addition to Table 2, Table 3, Table 4 and Table 5, which present the selected bond lengths, bond angles, torsion angles, hydrogen bond distances, and angles, respectively. The crystallographic data have been deposited with the Cambridge Crystallographic Data Centre as the supplementary publication number is 2150435 for **VK3a**. Copies of the data can be obtained free of charge on application to CCDC, 12 Union Road, Cambridge CB2 1EZ, UK [fax: +44(1223)336033, e-mail: deposit@ccdc.cam.ac.uk].

### 3.3. General Procedure for the Synthesis of the Thiolated Vitamin K3 (**VK3**) Analogs

To a stirred solution of **VK3** (100 mg, 1 eq.) in ethanol (20 mL) was added corresponding thiol (1 eq.) dropwise at room temperature. The reaction mixture was then refluxed until the consumption of the starting material as reported in the literature [47]. The reaction mixture was cooled to an ambient temperature. After the reaction mixture was concentrated under reduced pressure, the residue was dissolved with CH_2_Cl_2_ (50 mL), and the solution was washed sequentially with water (3 × 30 mL). The organic layer was dried over CaCl_2_, filtered, and concentrated under reduced pressure, and the residue was purified by means of column chromatography on silica gel to afford the desired products (**VK3** analogs).

#### 3.3.1. Methyl 2-(3-Methyl-1,4-dioxo-1,4-dihydronaphthalen-2-ylthio)acetate (**VK3a**)

The general procedure was followed using methyl thioglycolate (68 mg, 1 eq.) and **VK3**. Purification by column chromatography on silica gel (PET/EtAc, *v*/*v* 4:1) yielded **VK3a** (75%) [9] as a yellow crystal, mp 92.5–93.3 °C. FTIR (ATR) υ (cm^−1^): 2944, 2852 (CH_aliphatic_), 1721, 1660, 1588, 1562 (>C=O), 1435, 1368, 1289, 1265, 1184, 1112, 1001; ^1^H NMR (500 MHz, *CDCl_3_*) *δ* (ppm): 8.14–7.98 (m, 2H, CH_aromatic_), 7.76–7.62 (m, 2H, CH_aromatic_), 4.00 (s, 2H, SCH_2_), 3.71 (s, 3H, OCH_3_), 2.38 (s, 3H, CH_3_); ^13^C NMR (125 MHz, *CDCl_3_*) *δ* (ppm): 182.1, 181.1, 169.7 (>C=O), 147.1, 144.5, 133.7, 133.4, 132.7, 132.0, 126.8, 126.6 (CH and C_q_), 52.6 (OCH_3_), 35.0 (SCH_2_), 15.1 (CH_3_); HRMS (TOF MS ES+) *m*/*z* calcd for C_14_H_13_O_4_S [M + H]^+^: 277.0535; found: 277.0535.

#### 3.3.2. Ethyl 2-(3-Methyl-1,4-dioxo-1,4-dihydronaphthalen-2-ylthio)acetate (**VK3b**)

The general procedure was followed using ethyl thioglycolate (77 mg, 1 eq.) and **VK3**. Purification by column chromatography on silica gel (PET/EtAc, *v*/*v* 4:1) yielded **VK3b** (70%) [76] as a yellow crystal, 68.6–70.2 °C. FTIR (ATR) υ (cm^−1^): 2985, 2914 (CH_aliphatic_), 1732, 1661, 1639 (>C=O), 1587, 1548, 1455, 1390, 1310, 1283, 1206, 1150, 1115, 1021; ^1^H NMR (500 MHz, *CDCl_3_*) *δ* (ppm): 8.21–7.94 (m, 2H, CH_aromatic_), 7.86–7.58 (m, 2H, CH_aromatic_), 4.17–4.08 (m, 2H, OCH_2_), 3.98 (s, 2H, SCH_2_), 2.39 (s, 3H, CH_3_), 1.20 (t, *J* = 7.1 Hz, 3H, CH_3_); ^13^C NMR (125 MHz, *CDCl_3_*) *δ* (ppm): 182.1, 181.1, 169.1 (>C=O), 147.1, 144.8, 133.7, 133.4, 132.8, 132.0, 126.8, 126.6 (CH and C_q_), 61.6 (OCH_2_), 35.2 (SCH_2_), 15.1, 14.1 (CH_3_); HRMS (TOF MS ES+) *m*/*z* calcd for C_15_H_15_O_4_S [M + H]^+^: 291.0691; found: 291.0707; calcd for C_15_H_14_O_4_SNa [M + Na]^+^: 313.0511; found: 313.0511.

#### 3.3.3. Ethyl 2-(3-Methyl-1,4-dioxo-1,4-dihydronaphthalen-2-ylthio)propanoate(**VK3c**)

The general procedure was followed using ethyl 2-mercaptopropanoate (86 mg, 1 eq.) and **VK3**. Purification by column chromatography on silica gel (PET/EtAc, *v*/*v* 4:1) yielded **VK3c** (92%) as a yellow oil. FTIR (ATR) υ (cm^−1^): 2981, 2933 (CH_aliphatic_), 1732, 1661, 1592 (>C=O), 1568, 1448, 1370, 1320, 1280, 1250, 1158, 1062, 1021; ^1^H NMR (500 MHz, *CDCl_3_*) *δ* (ppm): 8.26–7.92 (m, 2H, CH_aromatic_), 7.82–7.57 (m, 2H, CH_aromatic_), 4.70–4.35 (m, 1H, SCH), 4.30–3.89 (m, 2H, OCH_2_), 2.38 (s, 3H, CH_3_), 1.58 (d, *J* = 7.2 Hz, 3H, CH_3_), 1.08 (t, *J* = 7.1 Hz, 3H, CH_3_); ^13^C NMR (125 MHz, *CDCl_3_*) *δ* (ppm): 182.5, 181.0, 172.0 (>C=O), 148.1, 145.4, 133.7, 133.5, 132.8, 132.0, 126.9, 126.6 (CH and C_q_), 61.3 (OCH_2_), 43.3 (SCH), 16.9, 15.4, 14.0 (CH_3_); HRMS (TOF MS ES+) *m*/*z* calcd for C_16_H_17_O_4_S [M + H]^+^: 305.0848; found: 305.0849.

#### 3.3.4. Methyl 3-(3-Methyl-1,4-dioxo-1,4-dihydronaphthalen-2-ylthio)propanoate (**VK3d**)

The general procedure was followed using methyl 3-mercaptopropanoate (77 mg, 1 eq.) and **VK3**. Purification by column chromatography on silica gel (PET/EtAc, *v*/*v* 4:1) yielded **VK3d** (62%) [77] as a yellow crystal, 76.2–77.9 °C. FTIR (ATR) υ (cm^−1^): 2996, 2951, 2848 (CH_aliphatic_), 1717, 1666, 1649 (>C=O), 1590, 1561, 1436, 1370, 1317, 1280, 1245, 1181, 1108; ^1^H NMR (500 MHz, *CDCl_3_*) *δ* (ppm): 8.20–7.98 (m, 2H, CH_aromatic_), 7.79–7.62 (m, 2H, CH_aromatic_), 3.69 (s, 3H, OCH_3_), 3.46 (t, *J* = 7.0 Hz, 2H, SCH_2_), 2.72 (t, *J* = 6.9 Hz, 2H, CH_2_), 2.36 (s, 3H, CH_3_); ^13^C NMR (125 MHz, *CDCl_3_*) *δ* (ppm): 182.2, 181.2, 171.9 (>C=O), 147.4, 145.8, 133.7, 133.4, 132.8, 132.0, 126.8, 126.6 (CH and C_q_), 51.9 (OCH_2_), 35.5, 29.3 (SCH_2_CH_2_), 15.3 (CH_3_); HRMS (TOF MS ES+) *m*/*z* calcd for C_15_H_15_O_4_S [M + H]^+^: 291.0691; found: 291.0691; calcd for C_15_H_14_O_4_SNa [M + Na]^+^: 313.0511; found: 313.0512.

#### 3.3.5. Ethyl 3-(3-Methyl-1,4-dioxo-1,4-dihydronaphthalen-2-ylthio)propanoate (**VK3e**)

The general procedure was followed using ethyl 3-mercaptopropanoate (86 mg, 1 eq.) and **VK3**. Purification by column chromatography on silica gel (PET/EtAc, *v*/*v* 4:1) yielded **VK3e** (95%) as a yellow oil. FTIR (ATR) υ (cm^−1^): 2979, 2904 (CH_aliphatic_), 1726, 1661 (>C=O), 1589, 1553, 1481, 1411, 1371, 1340, 1284, 1254, 1209, 1153, 1108, 1030, 1015; ^1^H NMR (500 MHz, *CDCl_3_*) *δ* (ppm): 8.17–7.95 (m, 2H, CH_aromatic_), 7.77–7.62 (m, 2H, CH_aromatic_), 4.34–3.95 (m, 2H, OCH_2_), 3.53–3.35 (m, 2H, SCH_2_), 2.76–2.55 (m, 2H, CH_2_(C=O)), 2.36 (s, 3H, CH_3_), 1.31–1.16 (m, 3H, CH_3_); ^13^C NMR (125 MHz, *CDCl_3_*) *δ* (ppm): 182.2, 181.1, 171.4 (>C=O), 147.4, 145.9, 133.7, 133.4, 132.8, 132.0, 126.8, 126.6 (CH and C_q_), 60.8 (OCH_2_CH_3_), 35.7 (SCH_2_), 29.3 (SCH_2_(C=O)), 15.3, 14.2 (CH_3_); HRMS (TOF MS ES+) *m*/*z* calcd for C_16_H_17_O_4_S [M + H]^+^: 305.0848; found: 305.0847; calcd for C_16_H_16_O_4_SNa [M + Na]^+^: 327.0667; found: 327.0673.

#### 3.3.6. Butyl 3-(3-Methyl-1,4-dioxo-1,4-dihydronaphthalen-2-ylthio)propanoate (**VK3f**)

The general procedure was followed using butyl 3-mercaptopropanoate (104 mg, 1 eq.) and **VK3**. Purification by column chromatography on silica gel (PET/EtAc, *v*/*v* 4:1) yielded **VK3f** (66%) as a yellow oil. FTIR (ATR) υ (cm^−1^): 2959, 2930, 2867 (CH_aliphatic_), 1731, 1661, 1592 (>C=O), 1559, 1459, 1420, 1322, 1280, 1248, 1180, 1032; ^1^H NMR (500 MHz, *CDCl_3_*) *δ* (ppm): 8.20–8.00 (m, 2H, CH_aromatic_), 7.83–7.62 (m, 2H, CH_aromatic_), 4.18–3.97 (m, 2H, OCH_2_), 3.52–3.33 (m, 2H, SCH_2_), 2.80–2.62 (m, 2H, CH_2_(C=O)), 2.36 (s, 3H, CH_3_), 1.69–1.51 (m, 2H, CH_2_), 1.44–1.18 (m, 2H, CH_2_), 1.03–0.76 (m, 3H, CH_3_); ^13^C NMR (125 MHz, *CDCl_3_*) *δ* (ppm): 182.2, 181.2, 171.5 (>C=O), 147.4, 145.8, 133.7, 133.4, 132.8, 132.0, 126.8, 126.6 (CH and C_q_), 64.7 (OCH_2_), 35.7 (SCH_2_), 30.6 (SCH_2_(C=O)), 29.3, 19.1 (CH_2_), 15.3, 13.7 (CH_3_); HRMS (TOF MS ES+) *m*/*z* calcd for C_18_H_21_O_4_S [M + H]^+^: 333.1161; found: 333.1170; calcd for C_18_H_20_O_4_SNa [M + Na]^+^: 355.0980; found: 355.0988.

#### 3.3.7. 2-Ethylhexyl 2-(3-Methyl-1,4-dioxo-1,4-dihydronaphthalen-2-ylthio)acetate (**VK3g**)

The general procedure was followed using 2-ethylhexyl thioglycolate (131 mg, 1 eq.) and **VK3**. Purification by column chromatography on silica gel (PET/EtAc, *v*/*v* 4:1) yielded **VK3g** (72%) as a yellow oil. FTIR (ATR) υ (cm^−1^): 2959, 2929, 2863 (CH_aliphatic_), 1732, 1661, 1592 (>C=O), 1564, 1461, 1320, 1280, 1159, 1032; ^1^H NMR (500 MHz, *CDCl_3_*) *δ* (ppm): 8.21–7.88 (m, 2H, CH_aromatic_), 7.86–7.60 (m, 2H, CH_aromatic_), 4.26–3.89 (m, 4H, OCH_2_+SCH_2_), 2.38 (s, 3H, CH_3_), 1.54–1.43 (m, 1H, CH), 1.35–1.09 (m, 8H, CH_2_), 0.86 (t, *J* = 6.7 Hz, 3H, CH_3_), 0.80 (t, *J* = 7.4 Hz, 3H, CH_3_); ^13^C NMR (125 MHz, *CDCl_3_*) *δ* (ppm): 182.0, 181.1, 169.4 (>C=O), 146.8, 144.8, 133.7, 133.4, 132.8, 132.0, 126.8, 126.6 (CH and C_q_), 68.0 (OCH_2_), 38.7 (SCH_2_), 35.2 (CH), 30.2, 28.8, 23.6, 22.9 (CH_2_), 15.1, 14.0, 10.9 (CH_3_); HRMS (TOF MS ES+) *m*/*z* calcd for C_21_H_27_O_4_S [M + H]^+^: 375.1630; found: 375.1630.

### 3.4. Biological Evaluation

#### 3.4.1. MIC Evaluation

MICs of the molecules were examined by the broth microdilution technique approved by the Clinical and Laboratory Institute (CLSI) [48,49]. In total, ten different standard ATCC isolates were prepared according to the CLSI recommendations. The stock solutions of the tested molecules were prepared in DMSO. Serial two-fold dilutions ranging from 1250 to 0.06 μg/mL were prepared in Mueller Hinton Broth for the tested bacteria and an RPMI-1640 medium for the yeast, respectively.

According to the antimicrobial activity results, we aimed to identify in vitro activities of the **VK3a** and **VK3b** against clinically obtained strains by the broth microdilution dilution technique, as described by the CLSI recommendations [48,49]. For this assay, 20 nonduplicates, nosocomially acquired Methicillin-Resistant *Staphylococcus aureus* isolated from blood specimens between April and September 2017 were obtained from the Department of Infectious Diseases and Clinical Microbiology, Faculty of Medicine, Istanbul Medipol University. All strains were identified using API STAPH (bioM’erieux). Then, all the tested *S. aureus* isolates were chosen by using Oxacillin susceptibility to determine the Methicillin resistant isolates, approved by CLSI (MIC ≥ 4 µg/mL) [48]. The MIC was defined as the lowest concentration of tested extracts, giving complete inhibition of visible growth. Experiments were performed in triplicate. MBCs were determined at the end of the incubation period by removing two 0.01 mL samples from each well demonstrating no visible growth and plated onto TSA. Resultant colonies were counted after an overnight incubation at 37 °C. The MBC was defined as the lowest concentration of molecules giving at least a 99.9% killing of the initial inoculums [78].

#### 3.4.2. Determination of Time-Kill Curves

For the evaluation of the bactericidal activity of the selected molecules (**VK3a** and **VK3b**), the time-kill curve (TKC) method was performed at one and four times the MIC against one (1) *MRSA* clinical strains. Molecule-free controls were included for the tested strain. The inocula were quantified spectrophotometrically and added to the flasks to yield a final concentration of 1 × 10^6^ CFU/mL. The test tubes containing MHB with and without (growth control) molecules in a final volume of 10 mL were incubated in a 37 °C calibrated shaking water bath, and viable counts were determined at 0, 2, 4, 6, and 24 h intervals after inoculation, by subculturing 0.1 mL serial dilutions onto TSA plates. All tests were performed in duplicate. The lower limit of detection for the time-kill assay was 1log_10_ CFU/mL. Bactericidal activity was defined as a ≥3 log_10_ CFU/mL decrease from the initial inoculum.

#### 3.4.3. Determination of the Antibiofilm Activities

Biofilm attachment and inhibition of biofilm formation assays were performed by the previously described method with some modifications [36]. For biofilm attachment, an overnight culture of strong, biofilm-producing clinically *MRSA* isolate was made, which was diluted 1/50 to obtain 1 × 10^6^–1 × 10^7^ CFU/200 mL for bacteria in TSB supplemented with 1% glucose. Then the strain was added to each well of 96-well tissue culture microtiter plates with 1/10× MIC of tested molecules. The plates were allowed to incubate for 1, 2, and 4 h at 37 °C. The positive control was the studied strain using only the medium. After incubation, each well was washed with PBS solution three times and measured at OD 595 nm.

For inhibition of the biofilm formation, the tested strain was incubated in its medium containing the molecule at 1× and 1/10× in addition to 1/100× MIC at 37 °C for 24 h in microtiter plates. Six wells were used for each molecule. The positive control was the tested strain in its medium without molecules. After incubation, each well was washed with PBS solution three times and measured at OD 595 nm.

#### 3.4.4. Hemolysis Assay

Hemolytic activities of the **VK3a** and **VK3b** analogs were detected as previously reported with some modifications [79]. In brief, 2% human red erythrocyte suspension was prepared with PBS. Then, 1, 2.5, 5, 10, 25, 50, 100, and 200 µg/mL **VK3a** and **VK3b** were added to the erythrocyte suspension. The positive control erythrocyte suspension was prepared with distilled water and the negative control was prepared in 1% DMSO. The samples were incubated at 37 °C for 1 h. After centrifugation at 1500 rpm for 5 min, the resulting supernatants were transferred to a 96-well plate and the absorbance was read at 405 nm using a microplate reader (Epoc, BioTek). The percent of the hemolysis was calculated as follows: Hemolysis % = [(OD_405nm(analogs)_ − OD_405nm(negative control)_)/(OD_405nm(positive control)_ − OD_405nm(negative control)_)].

### 3.5. Statistical Analysis

All experiments were performed in two independent assays. Two-way ANOVA-Tukey’s multiple comparison test was used to compare differences between control and antimicrobials treated biofilms. *p* value < 0.005 was considered as statistically significant.

### 3.6. Molecular Docking

TMK X-ray crystal structure (PDB code 4GFD) [58] was retrieved from Protein Data Bank to be utilized as a model in the present study. The protein structure was prepared using QuickPrep module of MOE (Version 2019.01, Chemical Computing Group Inc., Montreal, QC, Canada). Only one monomer, chain A, was selected and water residues were deleted. The docking study was conducted using the rigid-receptor method [80]. The co-crystallized ligand TK-666 was defined as the center of the binding site. Using the MOE build suite, the chemical structures were drawn, and then energy-minimized using the MOE default force field [81]. All other docking options were kept at their default values. Fifteen docking positions were generated for each ligand. The generated docking positions were visualized using MOE.

### 3.7. In Silico Drug-Likeness and ADMET Analysis

The online SwissADME database was used to predict the drug-likeness and ADME parameters [82].

## 4. Conclusions

Today, antimicrobial resistance is still a main health problem in the world. Although many antibiotic drugs with adverse effects are available, novel drugs with different activity mechanisms are needed as soon as possible. In summary, a series of the thiolated **VK3** analogs (**VK3a–g**) were designed and synthesized by a simple, fast, high-efficiency, and applicable method. In vitro antimicrobial activity assays were performed on seven different bacterial strains in addition to three different fungal strains by the serial broth microdilution method. The obtained results indicated that introducing alkyl chain thiols into the **VK3** core led to an improvement in the antibacterial activity of the **VK3** analogs. Of these, two thiolated **VK3** analogs (**VK3a** and **VK3b**) were also tested for additional studies to reveal the antimicrobial and safety profile. Furthermore, they were tested for understanding the mode of action with evaluation by the time-kill kinetic study. Furthermore, we carried out another study on two analogs, active on *S. aureus* and *E. faecalis*, via determining their biofilm inhibition capacities. The present study is the first record to evaluate the antibiofilm and bactericidal profile against clinically resistant species. In line with this, **VK3b** showed the most potent antimicrobial activity against the clinically obtained *MRSA* strains with 4.88 µg/mL MIC_50_ value. Therefore, in accordance with the time-kill curve study results, these two molecules, **VK3a** and **VK3b**, showed bacterial regrowth at 1× and 4× MIC concentrations used within 24 h against the Methicillin-Resistant *S. aureus* isolate, and these molecules decreased the viable cell count at 6 h. Therefore, to achieve synergistic activity, it would be possible to use these molecules in combination with conventional antibiotics, for reducing antibiotic toxicity and antimicrobial resistance. Furthermore, **VK3a** and **VK3b** had no obvious hemolytic activity. The molecular docking of **VK3a** and **VK3b** into *S. aureus* thymidylate kinase showed similar placement and interactions with critical amino acids. Their interesting leadlikeness and pharmacokinetics make them worthy of further attention. Although finding safe and effective antimicrobial drugs are quite difficult, it is indeed necessary to conduct these studies continuously, especially in the era of antimicrobial resistance.

## Figures and Tables

**Table 1:** Crystallographic data for the **VK3a**.

Identification Code	VK3a
Chemical formula	C_14_H_12_O_4_S
Formula weight (g mol^−1^)	276.30
Temperature (K)	273
Radiation λ (Å)	0.71073
Crystal system	Monoclinic
Space groups, Z	P 1 2_1_/n 1, 4
Unit cell dimensions (Å)	a = 17.1194(16)
	b = 3.9184(4)
	c = 19.3277(18)
	α, γ = 90° β = 101.4320(10)°
Volume (Å^3^)	1270.8(2)
Crystal sizes (mm)	0.087 × 0.143 × 0.360
dcalc (g cm^−3^)	1.444
Absorption coefficient (mm^−1^)	0.261
Absorption correction, Tmin, Tmax	none, 0.9780 and 0.9120
θ_max_, deg	1.77 to 27.48
Goodness-of-fit on F^2^	1.018
Index ranges	−22 ≤ h ≤ 22
	−5 ≤ k ≤ 5
	−25 ≤ l ≤ 25
Reflections collected	15,493
Independent reflections	2896 [R(int) = 0.0558]
Final R indices [I > 2σ(I)]	R1 = 0.0417
	wR2 = 0.1064
R indices (all data)	R1 = 0.0587
	wR2 = 0.1162
Refinement method	Full-matrix least-squares on F^2^
Data/restraints/parameters	2896/0/174
Largest diff. peak and hole (eÅ^−3^)	0.279 and −0.231

**Table 2:** Selected bond lengths (Å) for **VK3a**.

S1-C4	1.7583(18)	S1-C3	1.8145(18)
O2-C2	1.325(2)	O2-C1	1.449(2)
O3-C14	1.218(2)	O1-C2	1.195(2)
O4-C7	1.216(2)	C2-C3	1.500(3)

**Table 3:** Selected bond angles (°) for **VK3a**.

C4-S1-C3	102.70(8)	C2-O2-C1	116.90(16)
C5-C4-C14	121.34(16)	C5-C4-S1	119.68(13)
C14-C4-S1	118.61(12)	C12-C13-C8	119.37(17)
C4-C5-C6	123.16(17)	C7-C5-C6	116.23(15)
O1-C2-O2	124.19(18)	O1-C2-C3	122.32(18)
O2-C2-C3	113.48(16)	C2-C3-S1	112.18(12)

**Table 4:** Selected torsion angles (°) for **VK3a**.

C3-S1-C4-C5	137.42(16)	C3-S1-C4-C14	−49.49(16)
C1-O2-C2-O1	0.2(3)	C8-C13-C14-O3	−169.01(19)
C6-C5-C7-O4	0.8(3)	S1-C4-C14-O3	−5.4(3)
C9-C8-C7-O4	−1.6(3)	C1-O2-C2-C3	−178.98(18)
O1-C2-C3-S1	164.08(18)	O2-C2-C3-S1	−16.7(2)
C4-S1-C3-C2	−151.94(14)	C4-C5-C7-O4	179.9(2)

**Table 5:** Hydrogen-bond geometry (Å, °) for **VK3a**.

.D-H···A	D-H	H···A	D···A	D-H···A
C3-H3A···O3 ^i^	0.97	2.56	3.513 (2)	168

Symmetry code: ^i^ x, y−1, z.

**Table 6:** The minimal inhibitory concentration (MIC) value of the thiolated **VK3** analogs (**VK3a–g**) for antibacterial activity ^a^.

Thiolated VK3		Substituents		Gram-Negative Bacteria ^a^ (MIC, μg/mL)				Gram-Positive Bacteria ^b^ (MIC, μg/mL)		
General Formula	ID	R_1_	R_2_	Pa	Ec	Kp	Pm	Sa	Se	Ef
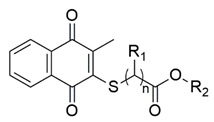	**VK3a** (n = 1)	H	-CH_3_	-	-	-	-	4.88	-	39.06
	**VK3b** (n = 1)	H	-CH_2_CH_3_	-	-	-	-	4.88	-	39.06
	**VK3c** (n = 1)	CH_3_	-CH_2_CH_3_	-	-	-	-	9.76	-	39.06
	**VK3d** (n = 2)	H	-CH_3_	-	-	-	-	4.88	-	39.06
	**VK3e** (n = 2)	H	-CH_2_CH_3_	-	-	-	-	312.50	-	-
	**VK3f** (n = 2)	H	-(CH_2_)_3_CH_3_	-	-	-	-	9.76	-	312.50
	**VK3g** (n = 1)	H	-CH_2_CH(n-C_2_H_5_)(CH_2_)_3_CH_3_	-	-	-	-	312.50	-	-
**Ceftazidime**				2.44						
**Cefuroxime-Na**					4.88	4.88	2.44	1.22		
**Cefuroxime**									9.76	
**Amikacin**										128.00

^a^ Gram-negative bacteria: *Pseudomonas aeruginosa* (ATCC 27853, **Pa**), *Escherichia coli* (ATCC 25922, **Ec**), *Klebsiella penumoniae* (ATCC 4352, **Kp**), and *Proteus mirabilis* (ATCC 14153, **Pm**). ^b^ Gram-positive bacteria: *Staphylococcus aureus* (ATCC 29213, **Sa**), *Staphylococcus epidermidis* (ATCC 12228, **Se**), and *Enterococcus faecalis* (ATCC 29212, **Ef**). “-” means no activity.

**Table 7:** The minimal inhibitory concentration (MIC) value of the thiolated **VK3** analogs (**VK3a–g**) for antifungal activity ^a^.

Thiolated VK3		Substituents		Fungi ^a^ (MIC, μg/mL)		
General Formula	ID	R_1_	R_2_	Ca	Cp	Ct
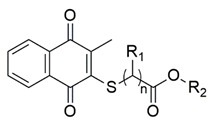	**VK3a** (n = 1)	H	-CH_3_	39.06	78.12	9.76
	**VK3b** (n = 1)	H	-CH_2_CH_3_	9.76	78.12	19.53
	**VK3c** (n = 1)	CH_3_	-CH_2_CH_3_	39.06	78.12	39.06
	**VK3d** (n = 2)	H	-CH_3_	39.06	156.25	9.76
	**VK3e** (n = 2)	H	-CH_2_CH_3_	-	-	-
	**VK3f** (n = 2)	H	-(CH_2_)_3_CH_3_	78.12	-	19.53
	**VK3g** (n = 1)	H	-CH_2_CH(n-C_2_H_5_)(CH_2_)_3_CH_3_	-	-	625
**Clotrimazole**				4.9		
**Amphotericin B**					0.5	1

^a^ Fungi: *Candida albicans* (ATCC 10231, **Ca**), *Candida parapsilosis* (ATCC 22019, **Cp**), and *Candida tropicalis* (ATCC 750, **Ct**). “-” means no activity.

**Table 8:** The MIC and MBC distributions of **VK3a** and **VK3b** against 20 clinically obtained Methicillin-Resistant *Staphylococcus aureus* isolates.

Molecules	Number of Strains MIC *(MBC)*										MIC Range	MIC_50_	MIC_90_	MBC Range	MBC_50_	MBC_90_
	Concentrations (µg/mL)															
	>625	625	312.50	156.25	78.12	39.06	19.53	9.76	4.88	2.44						
**VK3a**	- *(1)*	4 *(3)*	- *(1)*	1 *(4)*	1 *(3)*	10 *(6)*	2 *(1)*	2 *(1)*			9.76–625	39.06	625	9.76–>625	78.12	625
**VK3b**				- *(1)*	- *(1)*	1 *(4)*	1 *(2)*	6 *(4)*	8 *(6)*	4 *(2)*	2.44–39.06	4.88	9.76	2.44–156.25	9.76	39.06

**Table 9:** In silico drug-likeness and the ADME analysis of selected thiolated **VK3** analogs.

	Property/Rule	VK3a	VK3b
Physico-chemical properties and drug-likeness prediction	MW	276	290
	Log P	2.34	2.62
	TPSA Å^2^	85.74	85.74
	HBA	4	4
	HBD	0	0
	Lipiniski	Yes, 0 violation	Yes, 0 violation
	Ghose	Yes	Yes
	Veber	Yes	Yes
	Egan	Yes	Yes
	Muegge	Yes	Yes
	Leadlikeness	Yes	Yes
ADME Prediction	BBB permeability	No	No
	GI absorption	High	high
	Log *S*	−3.08	−3.31
	Solubility	Soluble	Soluble
	Bioavailability score	0.55	0.55
	CYP1A2, CYP2C19, CYP2C9	Yes	Yes
	CYP2D6, CYP3A4	No	No

## Data Availability

Data is contained within the article and supplementary material.

## Supplementary Materials

The following supporting information can be downloaded at: https://www.mdpi.com/article/10.3390/ph15050586/s1, Figure S1: The cell growth percentage (GP) of **VK3a**; Figure S2: The cell growth percentage (GP) of **VK3b**; Figure S3: The cell growth percentage (GP) of **VK3c**; Figure S4: The cell growth percentage (GP) of **VK3d**; Figure S5: The cell growth percentage (GP) of **VK3e**; Figure S6: The cell growth percentage (GP) of **VK3f**; Figure S7: The cell growth percentage (GP) of **VK3g**; Figure S8: Purity chromatogram of the **VK3a**; Figure S9: Purity chromatogram of the **VK3b**; Figure S10: Purity chromatogram of the **VK3c**; Figure S11: Purity chromatogram of the **VK3d**; Figure S12: Purity chromatogram of the **VK3e**; Figure S13: Purity chromatogram of the **VK3f**; Figure S14: Purity chromatogram of the **VK3g**; Figure S15: ^1^H NMR (500 MHz) spectrum of the **VK3a** in CDCl_3_-*d1*; Figure S16: ^13^C NMR (125 MHz) spectrum of the **VK3a** in CDCl_3_-*d1*; Figure S17: ^1^H NMR (500 MHz) spectrum of the **VK3b** in CDCl_3_-*d1*; Figure S18: ^13^C NMR (125 MHz) spectrum of the **VK3b** in CDCl_3_-*d1*; Figure S19: ^1^H NMR (500 MHz) spectrum of the **VK3c** in CDCl_3_-*d1*; Figure S20: ^13^C NMR (125 MHz) spectrum of the **VK3c** in CDCl_3_-*d1*; Figure S21: ^1^H NMR (500 MHz) spectrum of the **VK3d** in CDCl_3_-*d1*; Figure S22: ^13^C NMR (125 MHz) spectrum of the **VK3d** in CDCl_3_-*d1*; Figure S23: ^1^H NMR (500 MHz) spectrum of the **VK3e** in CDCl_3_-*d1*; Figure S24: ^13^C NMR (125 MHz) spectrum of the **VK3e** in CDCl_3_-*d1*; Figure S25: ^1^H NMR (500 MHz) spectrum of the **VK3f** in CDCl_3_-*d1*; Figure S26: ^13^C NMR (125 MHz) spectrum of the **VK3f** in CDCl_3_-*d1*; Figure S27: ^1^H NMR (500 MHz) spectrum of the **VK3g** in CDCl_3_-*d1*; Figure S28: ^13^C NMR (125 MHz) spectrum of the **VK3g** in CDCl_3_-*d1*; Table S1: Bond lengths (Å) for **VK3a**; Table S2: Bond angles (°) for **VK3a**; Table S3: Torsion angles (°) for **VK3a**.

## References

[1] Fairfield K.M., Fletcher R.H. (2002). Vitamins for chronic disease prevention in adults: Scientific review. JAMA.

[2] Rezaei H., Khiali S., Rezaei H., Rezaee H., Bannazadeh Baghi H., Pourghasem M., Entezari-Maleki T. (2021). Potential Roles of Vitamins in the Management of COVID-19: A Comprehensive Review. Pharm. Sci..

[3] Jovic T.H., Ali S.R., Ibrahim N., Jessop Z.M., Tarassoli S.P., Dobbs T.D., Holford P., Thornton C.A., Whitaker I.S. (2020). Could Vitamins Help in the Fight Against COVID-19?. Nutrients.

[4] Li X., Wang G., Chen D., Lu Y. (2015). β-Carotene and astaxanthin with human and bovine serum albumins. Food Chem..

[5] Bourassa P., Hasni I., Tajmir-Riahi H.A. (2011). Folic acid complexes with human and bovine serum albumins. Food Chem..

[6] Xu H., Liu Q., Zuo Y., Bi Y., Gao S. (2009). Spectroscopic Studies on the Interaction of Vitamin C with Bovine Serum Albumin. J. Solut. Chem..

[7] Haddad J.G. (1982). Human serum binding protein for vitamin D and its metabolites (DBP): Evidence that actin is the DBP binding component in human skeletal muscle. Arch. Biochem. Biophys..

[8] Dahlbäck B. (1983). Purification of human vitamin K-dependent protein S and its limited proteolysis by thrombin. Biochem. J..

[9] Suganthi M., Elango K.P. (2017). Synthesis, characterization and serum albumin binding studies of vitamin K3 derivatives. J. Photochem. Photobiol. B.

[10] Hamidi M.S., Cheung A.M. (2014). Vitamin K and musculoskeletal health in postmenopausal women. Mol. Nutr. Food Res..

[11] Scheiber D., Veulemans V., Horn P., Chatrou M.L., Potthoff S.A., Kelm M., Schurgers L.J., Westenfeld R. (2015). High-Dose Menaquinone-7 Supplementation Reduces Cardiovascular Calcification in a Murine Model of Extraosseous Calcification. Nutrients.

[12] Yerramsetti N., Dampanaboina L., Mendu V., Battula S. (2020). Synergistic factors ensue high expediency in the synthesis of menaquinone [K-2] analogue MK-6: Application to access an efficient one-pot protocol to MK-9. Tetrahedron.

[13] Li X.Y., Himes R.A., Prosser L.C., Christie C.F., Watt E., Edwards S.F., Metcalf C.S., West P.J., Wilcox K.S., Chan S.S.L. (2020). Discovery of the First Vitamin K Analogue as a Potential Treatment of Pharmacoresistant Seizures. J. Med. Chem..

[14] Wellington K.W., Hlatshwayo V., Kolesnikova N.I., Saha S.T., Kaur M., Motadi L.R. (2020). Anticancer activities of vitamin K3 analogues. Investig. New Drug.

[15] Doroshow J.H. (2020). Effect of Anticancer Quinones on Reactive Oxygen Production by Adult Rat Heart Myocytes. Oxidative Med. Cell. Longev..

[16] Bauer H., Fritz-Wolf K., Winzer A., Kuhner S., Little S., Yardley V., Vezin H., Palfey B., Schirmer R.H., Davioud-Charvet E. (2006). A fluoro analogue of the menadione derivative 6-[2′-(3′-methyl)-1′,4′-naphthoquinolyl]hexanoic acid is a suicide substrate of glutathione reductase. Crystal structure of the alkylated human enzyme. J. Am. Chem. Soc..

[17] Patel O.P.S., Beteck R.M., Legoabe L.J. (2021). Antimalarial application of quinones: A recent update. Eur. J. Med. Chem..

[18] Majiene D., Kuseliauskyte J., Stimbirys A., Jekabsone A. (2019). Comparison of the Effect of Native 1,4-Naphthoquinones Plumbagin, Menadione, and Lawsone on Viability, Redox Status, and Mitochondrial Functions of C6 Glioblastoma Cells. Nutrients.

[19] Deniz N.G., Abdassalam A.F.S., Ozyurek M., Yesil E.A., Sayil C. (2020). New vitamin K3 (menadione) analogues: Synthesis, characterization, antioxidant and catalase inhibition activities. J. Chem. Sci..

[20] Hileman E.O., Liu J.S., Albitar M., Keating M.J., Huang P. (2004). Intrinsic oxidative stress in cancer cells: A biochemical basis for therapeutic selectivity. Cancer Chemoth. Pharm..

[21] Tetef M., Margolin K., Ahn C., Akman S., Chow W., Leong L., Morgan R.J., Raschko J., Somlo G., Doroshow J.H. (1995). Mitomycin C and menadione for the treatment of lung cancer: A phase II trial. Investig. New Drugs.

[22] Osada S., Tomita H., Tanaka Y., Tokuyama Y., Tanaka H., Sakashita F., Takahashi T. (2008). The utility of vitamin K3 (menadione) against pancreatic cancer. Anticancer Res..

[23] Verrax J., Cadrobbi J., Marques C., Taper H., Habraken Y., Piette J., Calderon P.B. (2004). Ascorbate potentiates the cytotoxicity of menadione leading to an oxidative stress that kills cancer cells by a non-apoptotic caspase-3 independent form of cell death. Apoptosis.

[24] Suresh S., Raghu D., Karunagaran D. (2013). Menadione (Vitamin K3) Induces Apoptosis of Human Oral Cancer Cells and Reduces their Metastatic Potential by Modulating the Expression of Epithelial to Mesenchymal Transition Markers and Inhibiting Migration. Asian Pac. J. Cancer P.

[25] Ngo E.O., Sun T.P., Chang J.Y., Wang C.C., Chi K.H., Cheng A.L., Nutter L.M. (1991). Menadione-Induced DNA Damage in a Human Tumor-Cell Line. Biochem. Pharmacol..

[26] Zhang Z., Ding Y., Li J., Wang L., Xin X., Yan J., Wu J., Yuan A., Hu Y. (2021). Versatile iron-vitamin K3 derivative-based nanoscale coordination polymer augments tumor ferroptotic therapy. Nano Res..

[27] Bayrak N., Ciftci H.I., Yildiz M., Yildirim H., Sever B., Tateishi H., Otsuka M., Fujita M., Tuyun A.F. (2021). Structure based design, synthesis, and evaluation of anti-CML activity of the quinolinequinones as LY83583 analogs. Chem. Biol. Interact..

[28] Mataraci-Kara E., Bayrak N., Yildirim H., Yildiz M., Ataman M., Ozbek-Celik B., Tuyun A.F. (2021). Plastoquinone analogs: A potential antimicrobial lead structure intensely suppressing Staphylococcus epidermidis and Candida albicans growth. Med. Chem. Res..

[29] Jannuzzi A.T., Yıldız M., Bayrak N., Yıldırım H., Shilkar D., Jayaprakash V., TuYuN A.F. (2021). Anticancer agents based on Plastoquinone analogs with N-phenylpiperazine: Structure-activity relationship and mechanism of action in breast cancer cells. Chem.-Biol. Interact..

[30] Yildirim H. (2020). Arylthio analogs of 5,8-quinolinedione: Synthesis, characterization, antibacterial, and antifungal evaluations. Phosphorus Sulfur.

[31] Yildirim H. (2020). Synthesis, characterization, and biological evaluation of a set of new alkylthio substituted plastoquinones containing ester group. J. Mol. Struct..

[32] Ibis C., Tuyun A.F., Bahar H., Ayla S.S., Stasevych M.V., Musyanovych R.Y., Komarovska-Porokhnyavets O., Novikov V. (2013). Synthesis of novel 1,4-naphthoquinone derivatives: Antibacterial and antifungal agents. Med. Chem. Res..

[33] Ibis C., Tuyun A.F., Bahar H., Ayla S.S., Stasevych M.V., Musyanovych R.Y., Komarovska-Porokhnyavets O., Novikov V. (2014). Nucleophilic substitution reactions of 1,4-naphthoquinone and biologic properties of novel S-, S,S-, N-, and N,S-substituted 1,4-naphthoquinone derivatives. Med. Chem. Res..

[34] Espinosa-Bustos C., Vázquez K., Varela J., Cerecetto H., Paulino M., Segura R., Pizarro J., Vera B., González M., Zarate A.M. (2020). New aryloxy-quinone derivatives with promising activity on Trypanosoma cruzi. Arch. Pharm..

[35] De Almeida P.D.O., Jobim G.D.B., Ferreira C.C.D., Bernardes L.R., Dias R.B., Sales C.B.S., Valverde L.D., Rocha C.A.G., Soares M.B.P., Bezerra D.P. (2021). A new synthetic antitumor naphthoquinone induces ROS-mediated apoptosis with activation of the JNK and p38 signaling pathways. Chem.-Biol. Interact..

[36] Mataraci-Kara E., Bayrak N., Yildiz M., Yildirim H., TuYu N.A. (2021). Active Quinolinequinones against Methicillin-Resistant *Staphylococcus* spp.. Chem. Biodivers.

[37] Ciftci H.I., Bayrak N., Yıldız M., Yıldırım H., Sever B., Tateishi H., Otsuka M., Fujita M., Tuyun A.F. (2021). Design, synthesis and investigation of the mechanism of action underlying anti-leukemic effects of the quinolinequinones as LY83583 analogs. Bioorg. Chem..

[38] Bayrak N., Yildiz M., Yildirim H., Mataraci-Kara E., Tuyun A.F. (2021). Novel plastoquinone analogs containing benzocaine and its analogs: Structure-based design, synthesis, and structural characterization. Res. Chem. Intermediat..

[39] Bayrak N., Yildiz M., Yildirim H., Kara E.M., Celik B.O., Tuyun A.F. (2020). Brominated plastoquinone analogs: Synthesis, structural characterization, and biological evaluation. J. Mol. Struct..

[40] Wellington K.W., Kolesnikova N.I., Nyoka N.B.P., McGaw L.J. (2019). Investigation of the antimicrobial and anticancer activity of aminonaphthoquinones. Drug Dev. Res..

[41] Wellington K.W. (2015). Understanding cancer and the anticancer activities of naphthoquinones—A review. Rsc. Adv..

[42] Ryu C.K., Song A.L., Lee J.Y., Hong J.A., Yoon J.H., Kim A. (2010). Synthesis and antifungal activity of benzofuran-5-ols. Bioorg. Med. Chem. Lett..

[43] Ibis C., Ozsoy-Gunes Z., Tuyun A.F., Ayla S.S., Bahar H., Stasevych M.V., Musyanovych R.Y., Komarovska-Porokhnyavets O., Novikov V. (2016). Synthesis, antibacterial and antifungal evaluation of thio- or piperazinyl-substituted 1,4-naphthoquinone derivatives. J. Sulfur. Chem..

[44] Ravichandiran P., Maslyk M., Sheet S., Janeczko M., Premnath D., Kim A.R., Park B.H., Han M.K., Yoo D.J. (2019). Synthesis and Antimicrobial Evaluation of 1,4-Naphthoquinone Derivatives as Potential Antibacterial Agents. Chemistryopen.

[45] Ravichandiran P., Sheet S., Premnath D., Kim A.R., Yoo D.J. (2019). 1,4-Naphthoquinone Analogues: Potent Antibacterial Agents and Mode of Action Evaluation. Molecules.

[46] Ravichandiran P., Subramaniyan S.A., Kim S.Y., Kim J.S., Park B.H., Shim K.S., Yoo D.J. (2019). Synthesis and Anticancer Evaluation of 1,4-Naphthoquinone Derivatives Containing a Phenylaminosulfanyl Moiety. Chemmedchem.

[47] Tandon V.K., Maurya H.K., Tripathi A., ShivaKeshava G.B., Shukla P.K., Srivastava P., Panda D. (2009). 2,3-Disubstituted-1,4-naphthoquinones, 12H-benzo[b]phenothiazine-6, 11-diones and related compounds: Synthesis and Biological evaluation as potential antiproliferative and antifungal agents. Eur. J. Med. Chem..

[48] Clinical and Laboratory Standards Institute (CLSI) (2020). Performance Standards for Antimicrobial Susceptibility Testing.

[49] Clinical and Laboratory Standards Institute (CLSI) (1997). Reference Method for Broth Dilution Antifungal Susceptibility Testing of Yeasts; Approved Standard.

[50] Ricci M.S., Zong W.-X. (2006). Chemotherapeutic Approaches for Targeting Cell Death Pathways. Oncologist.

[51] Boyd M.R., Pauli K.D. (1995). Some Practical Considerations and Applications of the National-Cancer-Institute in-Vitro Anticancer Drug Discovery Screen. Drug Develop. Res..

[52] Razaghi A., Heimann K., Schaeffer P.M., Gibson S.B. (2018). Negative regulators of cell death pathways in cancer: Perspective on biomarkers and targeted therapies. Apoptosis.

[53] Da Silva R.E., Ribeiro F.d.O.S., de Carvalho A.M.A., Daboit T.C., Marinho-Filho J.D.B., Matos T.S., Pessoa O.D.L., de Souza de Almeida Leite J.R., de Araújo A.R., dos Santos Soares M.J. (2020). Antimicrobial and antibiofilm activity of the benzoquinone oncocalyxone A. Microb. Pathogenesis..

[54] Novais J.S., Carvalho M.F., Ramundo M.S., Beltrame C.O., Geraldo R.B., Jordao A.K., Ferreira V.F., Castro H.C., Figueiredo A.M.S. (2020). Antibiofilm effects of N,O-acetals derived from 2-amino-1,4-naphthoquinone are associated with downregulation of important global virulence regulators in methicillin-resistant Staphylococcus aureus. Sci. Rep.

[55] Mataraci-Kara E., Ozbek-Celik B. (2018). Investigation of the effects of various antibiotics against Klebsiella pneumoniae biofilms on in vitro catheter model. J. Chemotherapy.

[56] Crouzet M., Le Senechal C., Brözel V.S., Costaglioli P., Barthe C., Bonneu M., Garbay B., Vilain S. (2014). Exploring early steps in biofilm formation: Set-up of an experimental system for molecular studies. BMC Microbiol..

[57] Petit C.M., Koretke K.K. (2002). Characterization of Streptococcus pneumoniae thymidylate kinase: Steady-state kinetics of the forward reaction and isothermal titration calorimetry. Biochem. J..

[58] Keating T.A., Newman J.V., Olivier N.B., Otterson L.G., Andrews B., Boriack-Sjodin P.A., Breen J.N., Doig P., Dumas J., Gangl E. (2012). In Vivo Validation of Thymidylate Kinase (TMK) with a Rationally Designed, Selective Antibacterial Compound. Acs Chem. Biol..

[59] Kawatkar S.P., Keating T.A., Olivier N.B., Breen J.N., Green O.M., Guler S.Y., Hentemann M.F., Loch J.T., McKenzie A.R., Newman J.V. (2014). Antibacterial Inhibitors of Gram-Positive Thymidylate Kinase: Structure–Activity Relationships and Chiral Preference of a New Hydrophobic Binding Region. J. Med. Chem..

[60] Vanheusden V., Munier-Lehmann H., Froeyen M., Busson R., Rozenski J., Herdewijn P., Van Calenbergh S. (2004). Discovery of Bicyclic Thymidine Analogues as Selective and High-Affinity Inhibitors of Mycobacterium tuberculosis Thymidine Monophosphate Kinase. J. Med. Chem..

[61] Abo-Salem H.M., Abd El Salam H.A., Abdel-Aziem A., Abdel-Aziz M.S., El-Sawy E.R. (2021). Synthesis, Molecular Docking, and Biofilm Formation Inhibitory Activity of Bis(Indolyl)Pyridines Analogues of the Marine Alkaloid Nortopsentin. Molecules.

[62] Dechouk L.F., Bouchoucha A., Abdi Y., Si Larbi K., Bouzaheur A., Terrachet-Bouaziz S. (2022). Coordination of new palladium (II) complexes with derived furopyran-3,4-dione ligands: Synthesis, characterization, redox behaviour, DFT, antimicrobial activity, molecular docking and ADMET studies. J. Mol. Struct..

[63] Martínez-Botella G., Loch J.T., Green O.M., Kawatkar S.P., Olivier N.B., Boriack-Sjodin P.A., Keating T.A. (2013). Sulfonylpiperidines as novel, antibacterial inhibitors of Gram-positive thymidylate kinase (TMK). Bioorg. Med. Chem. Lett..

[64] Teague S.J., Davis A.M., Leeson P.D., Oprea T. (1999). The Design of Leadlike Combinatorial Libraries. Angew. Chem. Int. Ed. Engl..

[65] Lipinski C.A., Lombardo F., Dominy B.W., Feeney P.J. (2001). Experimental and computational approaches to estimate solubility and permeability in drug discovery and development settings. Adv. Drug Deliv. Rev..

[66] Ghose A.K., Viswanadhan V.N., Wendoloski J.J. (1999). A knowledge-based approach in designing combinatorial or medicinal chemistry libraries for drug discovery. 1. A qualitative and quantitative characterization of known drug databases. J. Comb. Chem..

[67] Veber D.F., Johnson S.R., Cheng H.-Y., Smith B.R., Ward K.W., Kopple K.D. (2002). Molecular Properties That Influence the Oral Bioavailability of Drug Candidates. J. Med. Chem..

[68] Egan W.J., Merz K.M., Baldwin J.J. (2000). Prediction of drug absorption using multivariate statistics. J. Med. Chem..

[69] Muegge I., Heald S.L., Brittelli D. (2001). Simple Selection Criteria for Drug-like Chemical Matter. J. Med. Chem..

[70] Bruker (2014). APEX2, Version 2014.1-1.

[71] Bruker (2013). SAINT, Version 8.34A.

[72] Bruker (2012). SADABS, Version 2012/2.

[73] Bruker (2000). SHELXTL, Version 6.14.

[74] Spek A.L. (2009). Structure validation in chemical crystallography. Acta Crystallogr. D.

[75] Macrae C.F., Edgington P.R., McCabe P., Pidcock E., Shields G.P., Taylor R., Towler M., van De Streek J. (2006). Mercury: Visualization and analysis of crystal structures. J. Appl. Crystallogr..

[76] Habib N.S., Mahran M.A. (2004). Synthesis and biological evaluation of novel naphthoquinone derivatives as potential anticancer and antimicrobial agents. Boll. Chim. Farm..

[77] Nagar B., Dhar B.B. (2022). Visible Light-Mediated Thiolation of Substituted 1,4-Naphthoquinones Using Eosin Y as a Photoredox Catalyst. J. Org. Chem..

[78] National Committee for Clinical Laboratory Standards (1999). Methods for Determining Bactericidal Activity of Antimicrobial Agents.

[79] Oddo A., Hansen P.R., Hansen P.R. (2017). Hemolytic Activity of Antimicrobial Peptides. Antimicrobial Peptides: Methods and Protocols.

[80] Tateishi H., Tateishi M., Radwan M.O., Masunaga T., Kawatashiro K., Oba Y., Oyama M., Inoue-Kitahashi N., Fujita M., Okamoto Y. (2021). A New Inhibitor of ADAM17 Composed of a Zinc-Binding Dithiol Moiety and a Specificity Pocket-Binding Appendage. Chem. Pharm. Bull..

[81] Mingle D., Ospanov M., Radwan M.O., Ashpole N., Otsuka M., Ross S.A., Walker L.A., Shilabin A.G., Ibrahim M.A. (2021). First in class (S,E)-11-[2-(arylmethylene)hydrazono]-PBD analogs as selective CB2 modulators targeting neurodegenerative disorders. Med. Chem. Res..

[82] Daina A., Michielin O., Zoete V. (2017). SwissADME: A free web tool to evaluate pharmacokinetics, drug-likeness and medicinal chemistry friendliness of small molecules. Sci. Rep..

