# Supplementary material for: Promising Antibacterial and Antifungal Agents Based on Thiolated Vitamin K3 Analogs: Synthesis, Bioevaluation, Molecular Docking
Source: Pharmaceuticals (Basel). 2022 May 10;15(5):586. doi: 10.3390/ph15050586 (PMC9146127; doi:10.3390/ph15050586)
Supplement: Supplementary file 1 [file pharmaceuticals-15-00586-s001.zip › pharmaceuticals-1666793-supplementary.pdf]

# Promising Antibacterial and Antifungal Agents Based on Thiolated Vitamin K3 Analogs: Synthesis, Bioevaluation, Molecular Docking

**Hatice Yıldırım [a], Mahmut Yıldız [b], Nilüfer Bayrak [a], Emel Mataracı-Kara [c], Mohamed O. Radwan [d,e], Ayse Tarbin Jannuzzi [f], Masami Otsuka [d,g], Mikako Fujita [d], and Amaç Fatih TuYuN *, [h]**

[a] *Department of Chemistry, Faculty of Engineering, Istanbul University-Cerrahpasa, Avcilar, 34320, Istanbul, Turkey*

[b] *Department of Chemistry, Gebze Technical University, Gebze, 41400, Kocaeli, Turkey*

[c] *Department of Pharmaceutical Microbiology, Faculty of Pharmacy, Istanbul University, Beyazit, 34116, Istanbul, Turkey*

[d] *Medicinal and Biological Chemistry Science Farm Joint Research Laboratory, Faculty of Life Sciences, Kumamoto University, 5–1 Oe-honmachi, Chuo-ku, Kumamoto, Kumamoto 862–0973, Japan*

[e] *Chemistry of Natural Compounds Department, Pharmaceutical and Drug Industries Research Division, National Research Centre, Dokki, Cairo 12622, Egypt*

[f] *Department of Pharmaceutical Toxicology, Faculty of Pharmacy, Istanbul University, Beyazit, Istanbul, Turkey*

[g] *Department of Drug Discovery, Science Farm Ltd., 1–7–30 Kuhonji, Chuo-ku, Kumamoto, Kumamoto 862–0976, Japan*

[h] *Department of Chemistry, Faculty of Science, Istanbul University, Fatih, Istanbul, Turkey*

* Author to whom correspondence should be addressed; E-Mail: aftuyun@gmail.com, aftuyun@istanbul.edu.tr (A. F. T.).

Tel.: +90212 440 0000.

## Contents


The cell growth percentage (GP) of the thiolated VK3 analogs (**VK3a-g**) S3-9

Purity chromatograms of the thiolated VK3 analogs (**VK3a-g**) S10-13

$^{1}$H and $^{13}$C spectra of the thiolated VK3 analogs (**VK3a-g**) S14-20

The details of all crystallographic data of the **VK3a** S21-23

## Developmental Therapeutics Program

## One Dose Mean Graph

| NSC: D-827958 / 1 | Conc: 1.00E-5 Molar | Test Date: Jan 25, 2021 |
|---|---|---|
| Experiment ID: 2101OS80 | | Report Date: May 06, 2021 |

| Panel/Cell Line | Growth Percent |
|---|---|
| **Leukemia** | |
| CCRF-CEM | 54.07 |
| HL-60(TB) | 45.79 |
| K-562 | 40.33 |
| MOLT-4 | 38.08 |
| RPMI-8226 | 46.82 |
| SR | 38.98 |
| **Non-Small Cell Lung Cancer** | |
| A549/ATCC | 104.10 |
| EKVX | 92.32 |
| HOP-62 | 100.29 |
| HOP-92 | 86.29 |
| NCI-H226 | 91.85 |
| NCI-H23 | 88.74 |
| NCI-H322M | 98.44 |
| NCI-H460 | 101.30 |
| NCI-H522 | 73.99 |
| **Colon Cancer** | |
| COLO 205 | 114.79 |
| HCC-2998 | 115.91 |
| HCT-116 | 83.04 |
| HCT-15 | 72.57 |
| HT29 | 112.90 |
| KM12 | 88.08 |
| SW-620 | 100.36 |
| **CNS Cancer** | |
| SF-268 | 96.12 |
| SF-295 | 104.02 |
| SF-539 | 72.09 |
| SNB-19 | 94.86 |
| SNB-75 | 85.14 |
| U251 | 93.03 |
| **Melanoma** | |
| LOX IMVI | 92.26 |
| MALME-3M | 110.85 |
| M14 | 101.63 |
| MDA-MB-435 | 104.92 |
| SK-MEL-2 | 116.35 |
| SK-MEL-28 | 107.76 |
| SK-MEL-5 | 96.79 |
| UACC-257 | 101.14 |
| UACC-62 | 80.15 |
| **Ovarian Cancer** | |
| IGROV1 | 97.71 |
| OVCAR-3 | 72.54 |
| OVCAR-4 | 74.54 |
| OVCAR-5 | 108.49 |
| OVCAR-8 | 93.13 |
| NCI/ADR-RES | 93.83 |
| SK-OV-3 | 104.83 |
| **Renal Cancer** | |
| 786-0 | 102.88 |
| A498 | 106.61 |
| ACHN | 76.44 |
| CAKI-1 | 71.04 |
| RXF 393 | 88.31 |
| SN12C | 87.96 |
| TK-10 | 141.03 |
| UO-31 | 64.35 |
| **Prostate Cancer** | |
| PC-3 | 79.16 |
| DU-145 | 111.75 |
| **Breast Cancer** | |
| MCF7 | 87.05 |
| MDA-MB-231/ATCC | 80.43 |
| HS 578T | 89.94 |
| BT-549 | 101.98 |
| T-47D | 79.44 |
| MDA-MB-468 | 58.44 |
| Mean | 88.63 |
| Delta | 50.55 |
| Range | 102.95 |

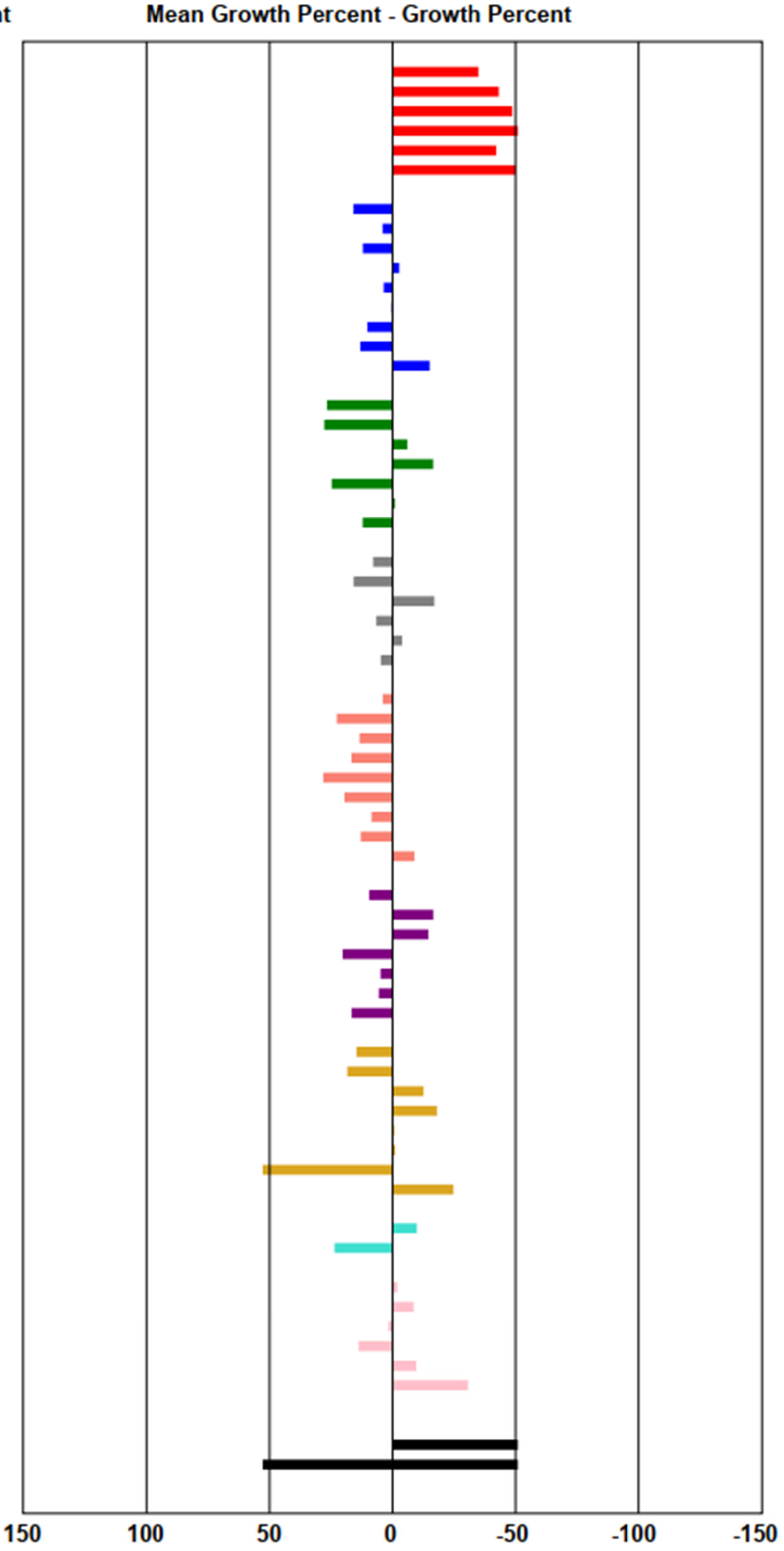


**Figure S1:** The cell growth percentage (GP) of **VK3a**

| Developmental Therapeutics Program<br>One Dose Mean Graph | NSC: D-827959 / 1 | Conc: 1.00E-5 Molar | Test Date: Jan 25, 2021 |
|---|---|---|---|
| | Experiment ID: 2101OS80 | | Report Date: May 06, 2021 |

| Panel/Cell Line | Growth Percent |
|---|---|
| Leukemia | |
| CCRF-CEM | 84.64 |
| HL-60(TB) | 59.32 |
| K-562 | 85.45 |
| MOLT-4 | 75.61 |
| RPMI-8226 | 92.43 |
| SR | 92.51 |
| Non-Small Cell Lung Cancer | |
| A549/ATCC | 107.97 |
| EKVX | 88.92 |
| HOP-62 | 104.49 |
| HOP-92 | 106.24 |
| NCI-H226 | 93.24 |
| NCI-H23 | 87.09 |
| NCI-H322M | 105.52 |
| NCI-H460 | 101.18 |
| NCI-H522 | 83.85 |
| Colon Cancer | |
| COLO 205 | 113.61 |
| HCC-2998 | 108.20 |
| HCT-116 | 100.09 |
| HCT-15 | 79.11 |
| HT29 | 108.18 |
| KM12 | 100.41 |
| SW-620 | 97.71 |
| CNS Cancer | |
| SF-268 | 100.28 |
| SF-295 | 101.15 |
| SF-539 | 95.08 |
| SNB-19 | 95.00 |
| SNB-75 | 92.67 |
| U251 | 97.11 |
| Melanoma | |
| LOX IMVI | 89.87 |
| MALME-3M | 115.79 |
| M14 | 106.18 |
| MDA-MB-435 | 108.79 |
| SK-MEL-2 | 108.21 |
| SK-MEL-28 | 110.42 |
| SK-MEL-5 | 102.11 |
| UACC-257 | 110.78 |
| UACC-62 | 93.33 |
| Ovarian Cancer | |
| IGROV1 | 104.61 |
| OVCAR-3 | 85.16 |
| OVCAR-4 | 92.00 |
| OVCAR-5 | 112.73 |
| OVCAR-8 | 99.26 |
| NCI/ADR-RES | 96.09 |
| SK-OV-3 | 108.63 |
| Renal Cancer | |
| 786-0 | 107.44 |
| A498 | 113.76 |
| ACHN | 91.41 |
| CAKI-1 | 80.71 |
| RXF 393 | 109.51 |
| SN12C | 96.85 |
| TK-10 | 131.99 |
| UO-31 | 71.41 |
| Prostate Cancer | |
| PC-3 | 89.59 |
| DU-145 | 114.09 |
| Breast Cancer | |
| MCF7 | 86.62 |
| MDA-MB-231/ATCC | 89.04 |
| HS 578T | 103.63 |
| BT-549 | 114.46 |
| T-47D | 102.45 |
| MDA-MB-468 | 88.68 |
| Mean | 98.21 |
| Delta | 38.89 |
| Range | 72.67 |

Mean Growth Percent - Growth Percent

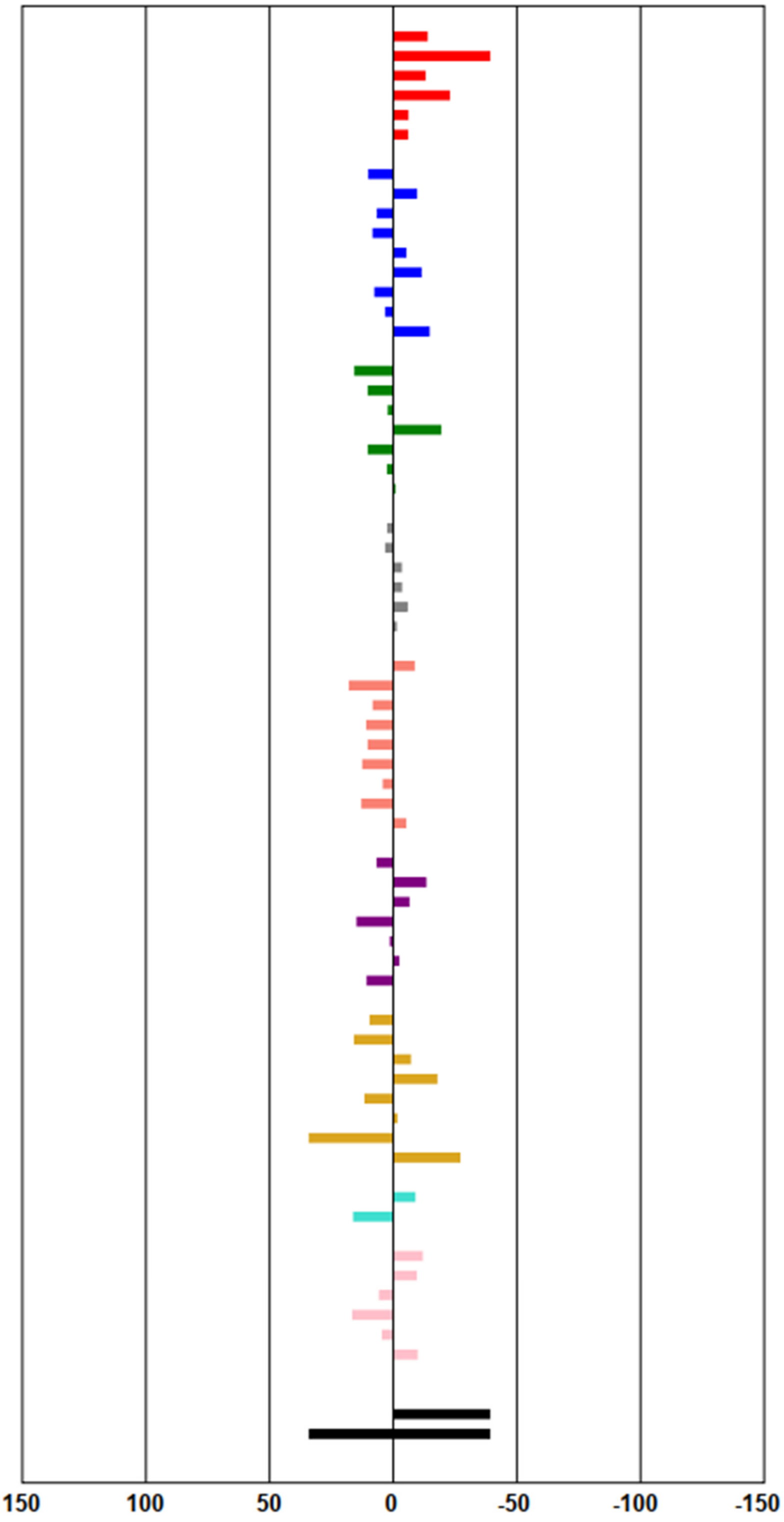


**Figure S2:** The cell growth percentage (GP) of **VK3b**

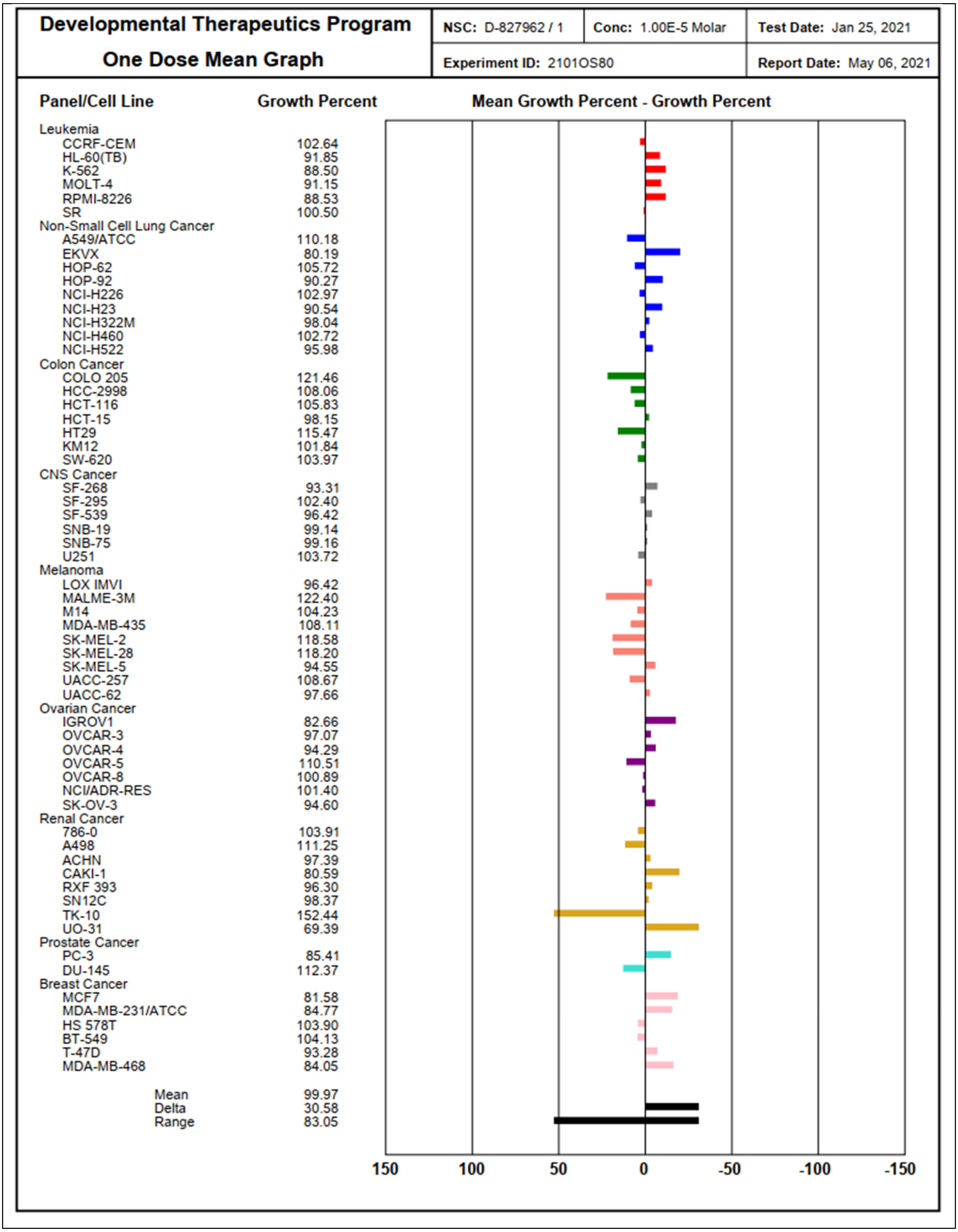


**Figure S3:** The cell growth percentage (GP) of **VK3c**

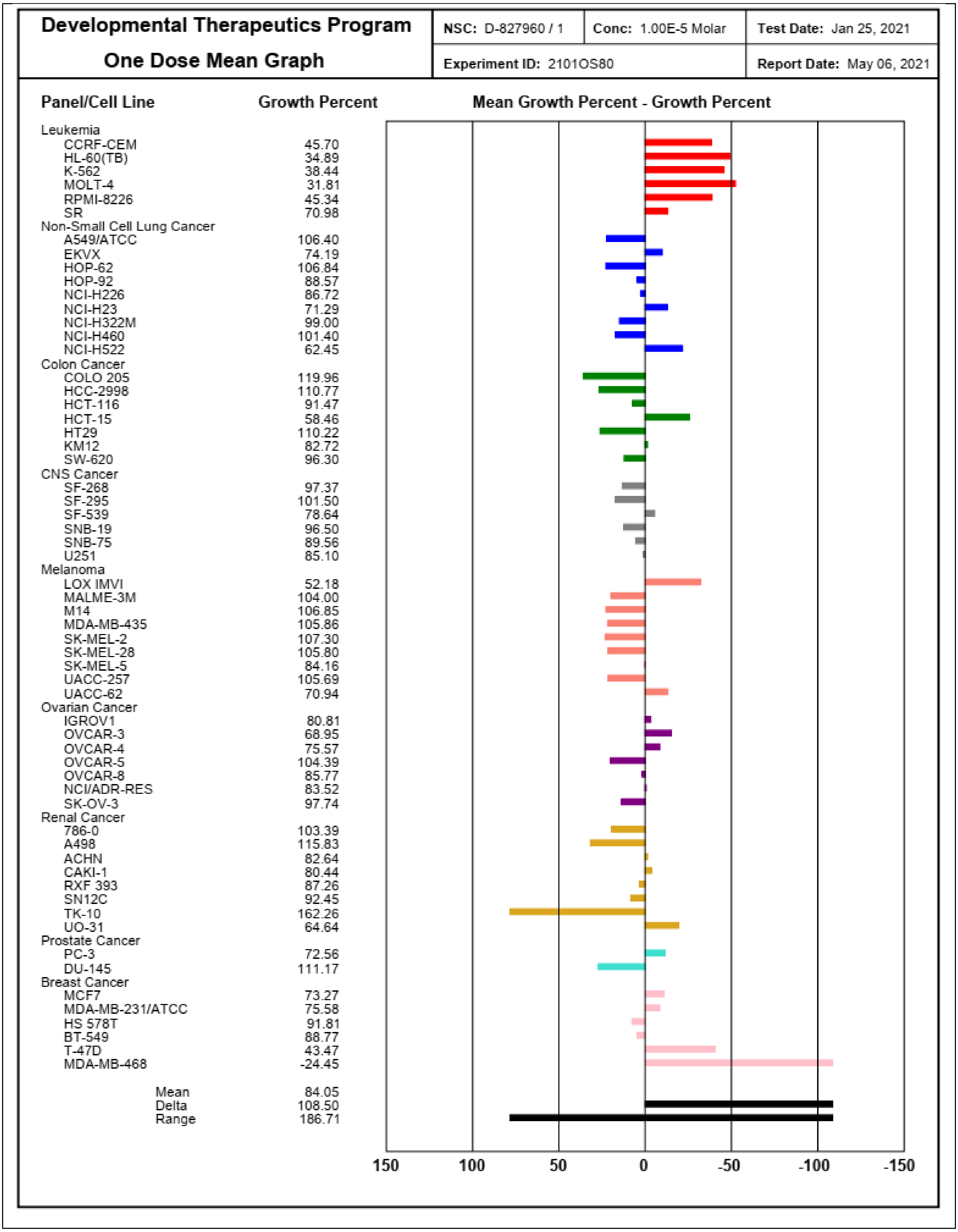


**Figure S4:** The cell growth percentage (GP) of **VK3d**

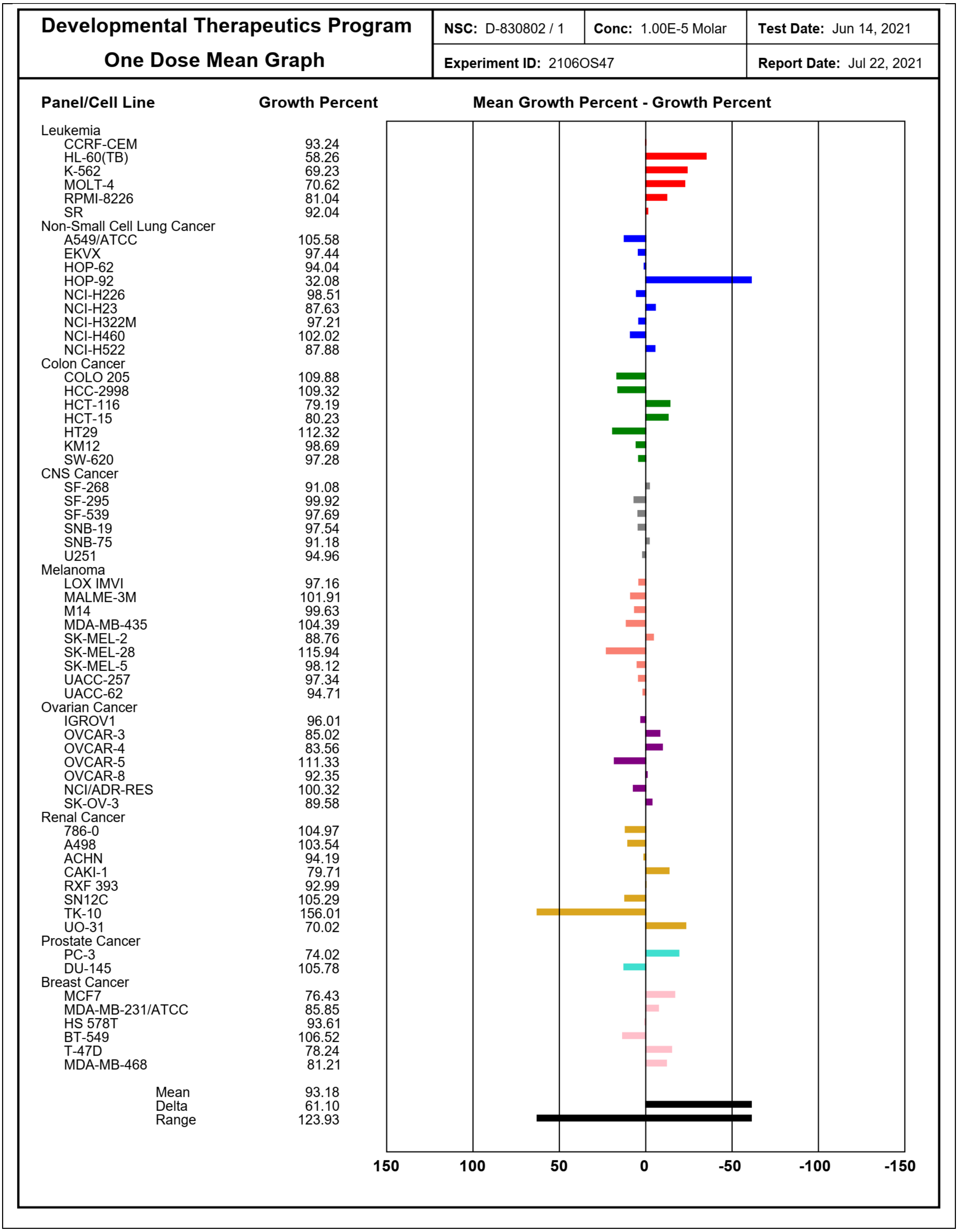


**Figure S5:** The cell growth percentage (GP) of **VK3e**

| Developmental Therapeutics Program One Dose Mean Graph | **NSC:** D-827961 / 1 | **Conc:** 1.00E-5 Molar | **Test Date:** Jan 25, 2021 |
|---|---|---|---|
| | **Experiment ID:** 2101OS80 | | **Report Date:** May 06, 2021 |

| Panel/Cell Line | Growth Percent |
|---|---|
| **Leukemia** | |
| CCRF-CEM | 97.40 |
| HL-60(TB) | 78.55 |
| K-562 | 73.63 |
| MOLT-4 | 79.08 |
| RPMI-8226 | 59.68 |
| SR | 86.01 |
| **Non-Small Cell Lung Cancer** | |
| A549/ATCC | 101.42 |
| EKVX | 87.48 |
| HOP-62 | 95.88 |
| HOP-92 | 81.14 |
| NCI-H226 | 91.18 |
| NCI-H23 | 89.70 |
| NCI-H322M | 99.23 |
| NCI-H460 | 101.75 |
| NCI-H522 | 69.40 |
| **Colon Cancer** | |
| COLO 205 | 117.54 |
| HCC-2998 | 106.86 |
| HCT-116 | 96.06 |
| HCT-15 | 83.35 |
| HT29 | 112.29 |
| KM12 | 91.14 |
| SW-620 | 102.91 |
| **CNS Cancer** | |
| SF-268 | 99.36 |
| SF-295 | 99.05 |
| SF-539 | 94.90 |
| SNB-19 | 98.45 |
| SNB-75 | 86.71 |
| U251 | 89.19 |
| **Melanoma** | |
| LOX IMVI | 87.17 |
| MALME-3M | 105.73 |
| M14 | 98.89 |
| MDA-MB-435 | 107.58 |
| SK-MEL-2 | 100.24 |
| SK-MEL-28 | 120.47 |
| SK-MEL-5 | 98.35 |
| UACC-257 | 102.30 |
| UACC-62 | 90.02 |
| **Ovarian Cancer** | |
| IGROV1 | 93.34 |
| OVCAR-3 | 84.01 |
| OVCAR-4 | 82.39 |
| OVCAR-5 | 115.38 |
| OVCAR-8 | 94.99 |
| NCI/ADR-RES | 99.42 |
| SK-OV-3 | 81.80 |
| **Renal Cancer** | |
| 786-0 | 101.40 |
| A498 | 99.84 |
| ACHN | 95.29 |
| CAKI-1 | 77.73 |
| RXF 393 | 100.50 |
| SN12C | 88.47 |
| TK-10 | 133.69 |
| UO-31 | 63.45 |
| **Prostate Cancer** | |
| PC-3 | 79.17 |
| DU-145 | 113.54 |
| **Breast Cancer** | |
| MCF7 | 80.05 |
| MDA-MB-231/ATCC | 87.12 |
| HS 578T | 97.96 |
| BT-549 | 105.95 |
| T-47D | 62.34 |
| MDA-MB-468 | 82.10 |
| Mean | 93.33 |
| Delta | 33.65 |
| Range | 74.01 |

**Mean Growth Percent - Growth Percent**

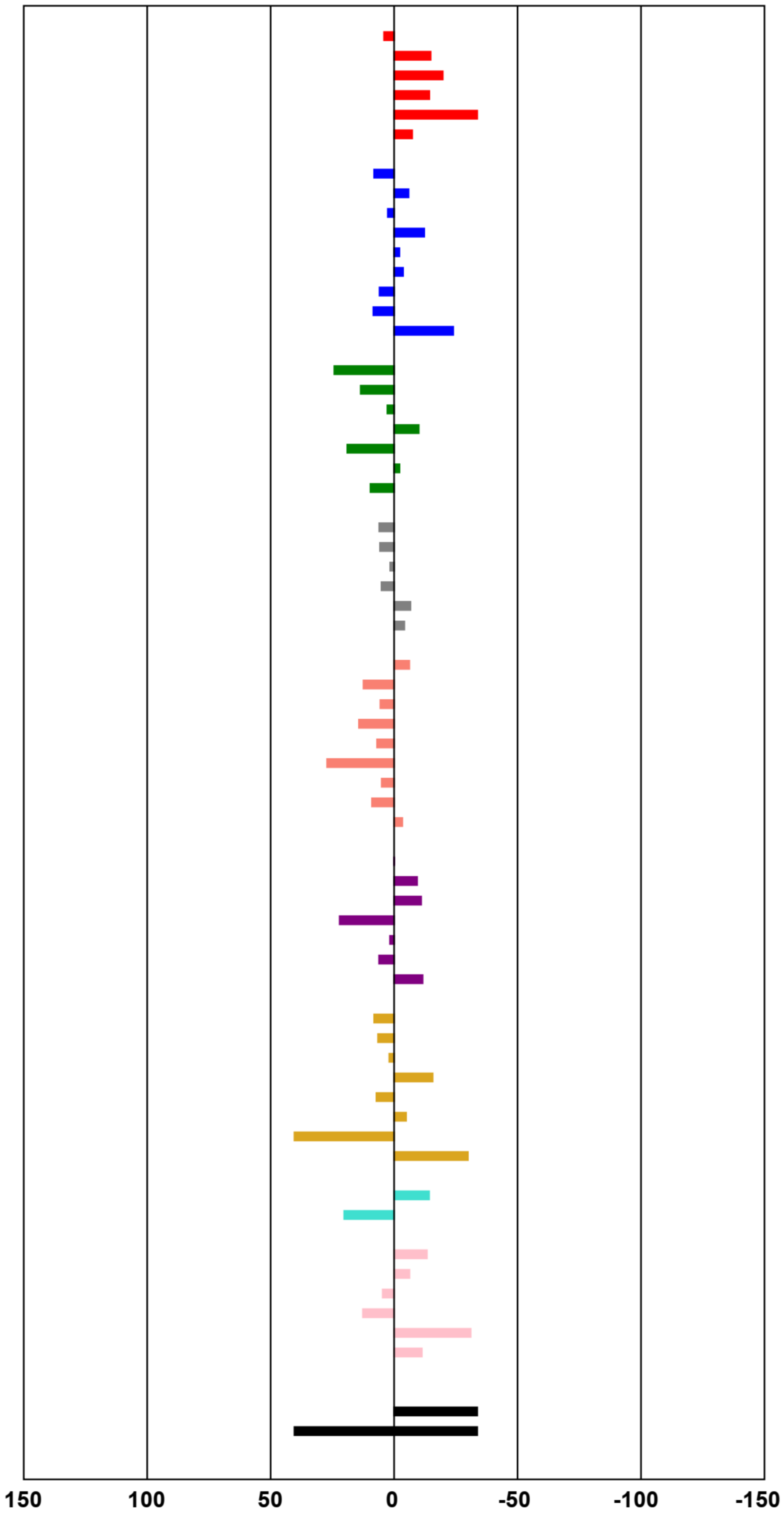


**Figure S6:** The cell growth percentage (GP) of **VK3f**

| Developmental Therapeutics Program<br>One Dose Mean Graph | NSC: D-827963 / 1 | Conc: 1.00E-5 Molar | Test Date: Jan 25, 2021 |
|---|---|---|---|
| | Experiment ID: 2101OS80 | | Report Date: May 06, 2021 |

| Panel/Cell Line | Growth Percent |
|---|---|
| **Leukemia** | |
| CCRF-CEM | 92.78 |
| HL-60(TB) | 78.30 |
| K-562 | 71.91 |
| MOLT-4 | 89.01 |
| RPMI-8226 | 56.72 |
| SR | 87.94 |
| **Non-Small Cell Lung Cancer** | |
| A549/ATCC | 107.10 |
| EKVX | 98.71 |
| HOP-62 | 108.59 |
| HOP-92 | 79.48 |
| NCI-H226 | 99.86 |
| NCI-H23 | 90.54 |
| NCI-H322M | 102.79 |
| NCI-H460 | 102.37 |
| NCI-H522 | 77.92 |
| **Colon Cancer** | |
| COLO 205 | 129.01 |
| HCC-2998 | 111.86 |
| HCT-116 | 87.32 |
| HCT-15 | 87.81 |
| HT29 | 122.27 |
| KM12 | 91.22 |
| SW-620 | 101.18 |
| **CNS Cancer** | |
| SF-268 | 99.37 |
| SF-295 | 103.98 |
| SF-539 | 87.37 |
| SNB-19 | 96.79 |
| SNB-75 | 87.00 |
| U251 | 97.43 |
| **Melanoma** | |
| LOX IMVI | 90.98 |
| MALME-3M | 120.73 |
| M14 | 96.30 |
| MDA-MB-435 | 103.14 |
| SK-MEL-2 | 114.37 |
| SK-MEL-28 | 109.21 |
| SK-MEL-5 | 96.64 |
| UACC-257 | 107.05 |
| UACC-62 | 84.72 |
| **Ovarian Cancer** | |
| IGROV1 | 98.02 |
| OVCAR-3 | 76.25 |
| OVCAR-4 | 74.12 |
| OVCAR-5 | 113.90 |
| OVCAR-8 | 97.14 |
| NCI/ADR-RES | 96.04 |
| SK-OV-3 | 112.11 |
| **Renal Cancer** | |
| 786-0 | 107.60 |
| A498 | 107.18 |
| ACHN | 90.34 |
| CAKI-1 | 85.62 |
| RXF 393 | 112.76 |
| SN12C | 92.57 |
| TK-10 | 147.80 |
| UO-31 | 61.68 |
| **Prostate Cancer** | |
| PC-3 | 72.04 |
| DU-145 | 113.51 |
| **Breast Cancer** | |
| MCF7 | 79.61 |
| MDA-MB-231/ATCC | 85.43 |
| HS 578T | 96.26 |
| BT-549 | 96.14 |
| T-47D | 90.02 |
| MDA-MB-468 | 61.05 |
| Mean | 95.62 |
| Delta | 38.90 |
| Range | 91.08 |

Mean Growth Percent - Growth Percent

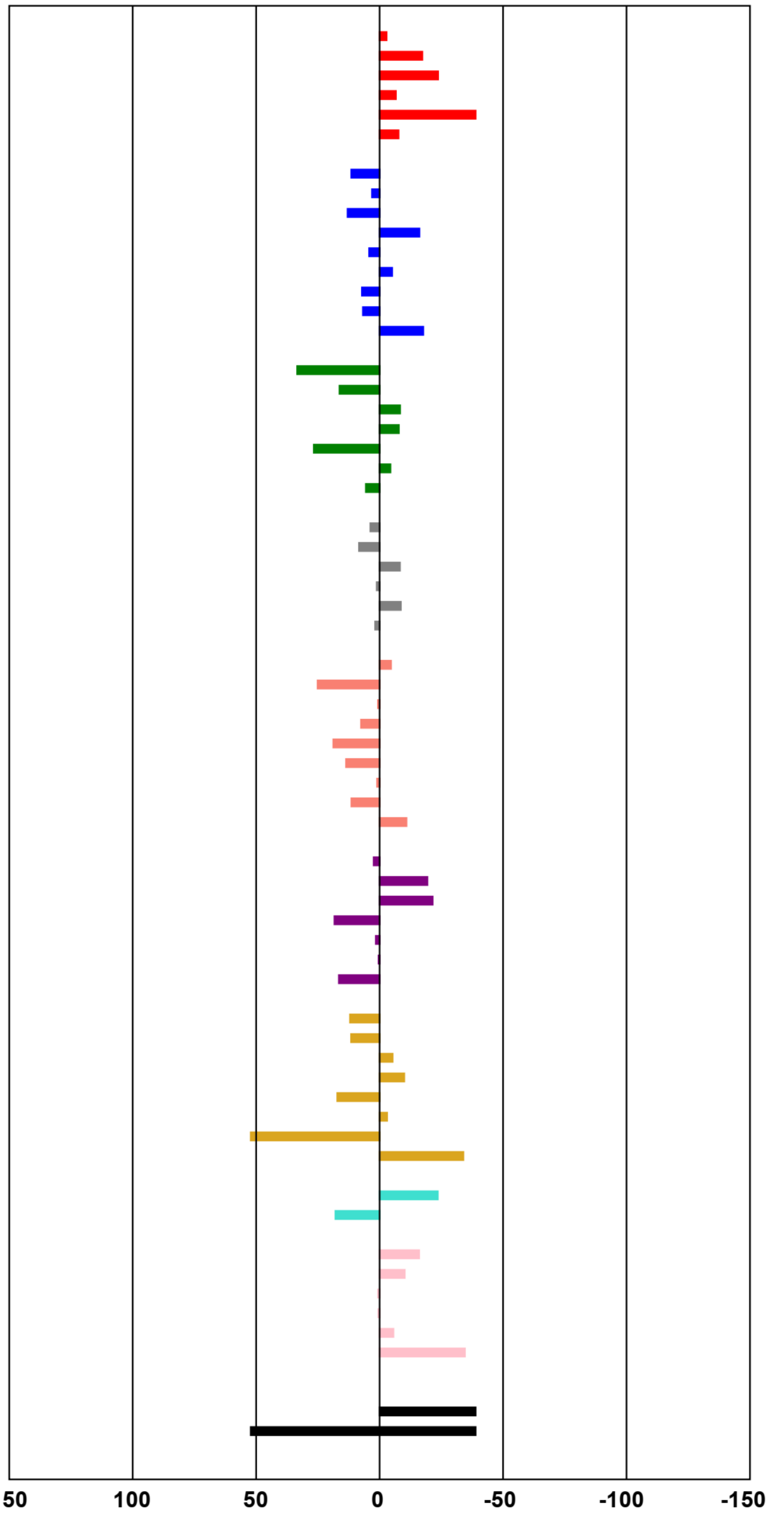


**Figure S7:** The cell growth percentage (GP) of **VK3g**

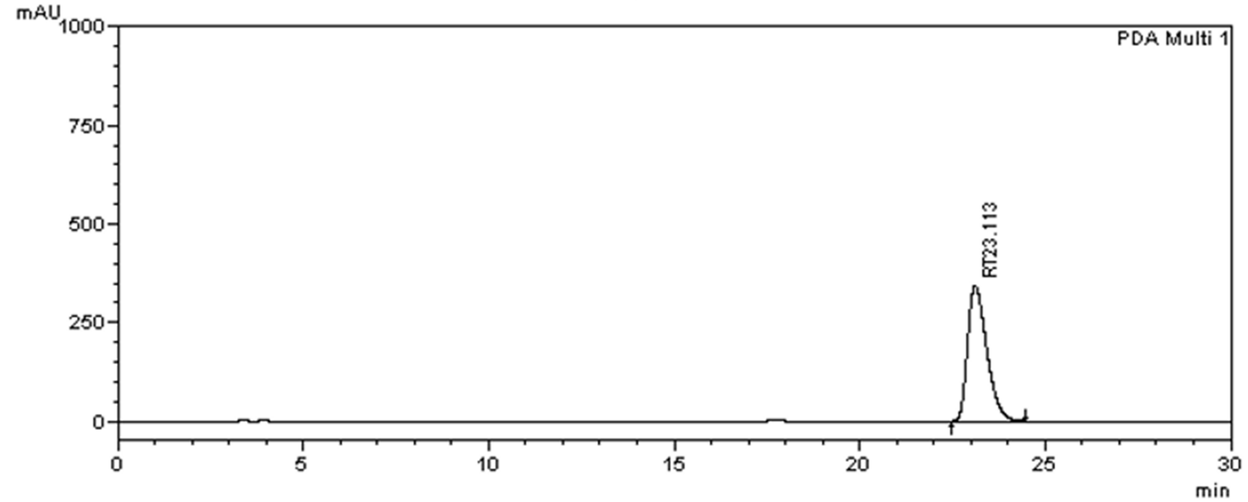


1 PDA Multi 1

<Results>

PDA Ch1 260nm 4nm

| Peak# | Ret. Time | Area | Height | Area % | Height % | Resolution |
|---|---|---|---|---|---|---|
| 1 | 23.113 | 12367573 | 343147 | 100.000 | 100.000 | 0.000 |
| Total | | 12367573 | 343147 | 100.000 | 100.000 | |

**Figure S8:** Purity chromatogram of the **VK3a**

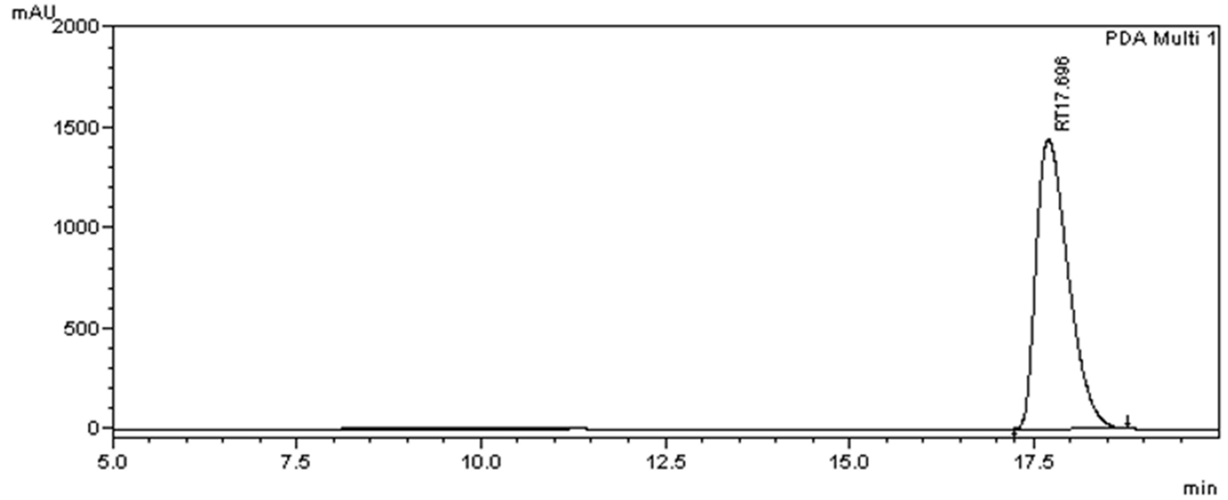


1 PDA Multi 1

<Results>

PDA Ch1 260nm 4nm

| Peak# | Ret. Time | Area | Height | Area % | Height % | Resolution |
|---|---|---|---|---|---|---|
| 1 | 17.696 | 43714001 | 1440955 | 100.000 | 100.000 | 0.000 |
| Total | | 43714001 | 1440955 | 100.000 | 100.000 | |

**Figure S9:** Purity chromatogram of the **VK3b**

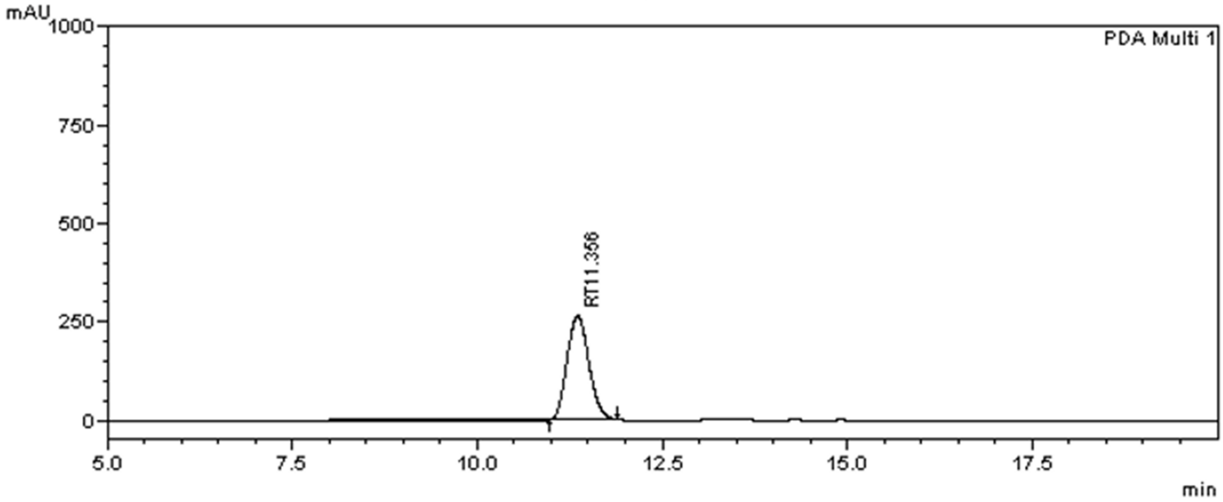


1 PDA Multi 1

<Results>

PDA Ch1 260nm 4nm

| Peak# | Ret. Time | Area | Height | Area % | Height % | Resolution |
|---|---|---|---|---|---|---|
| 1 | 11.356 | 5420450 | 263882 | 100.000 | 100.000 | 0.000 |
| Total | | 5420450 | 263882 | 100.000 | 100.000 | |

**Figure S10:** Purity chromatogram of the **VK3c**

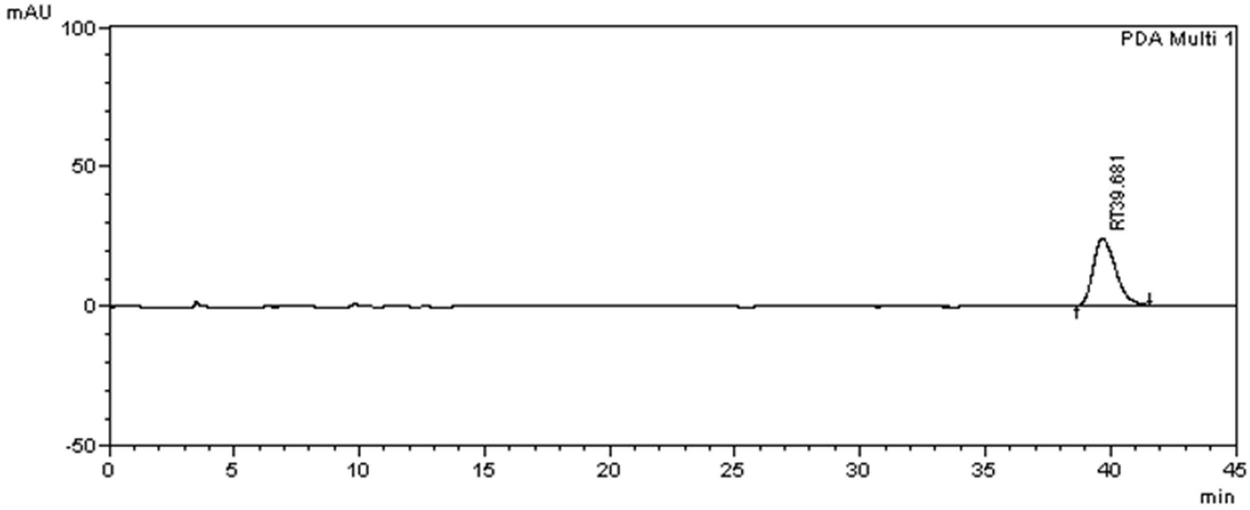


1 PDA Multi 1

<Results>

PDA Ch1 260nm 4nm

| Peak# | Ret. Time | Area | Height | Area % | Height % | Resolution |
|---|---|---|---|---|---|---|
| 1 | 39.681 | 1484849 | 24054 | 100.000 | 100.000 | 0.000 |
| Total | | 1484849 | 24054 | 100.000 | 100.000 | |

**Figure S11:** Purity chromatogram of the **VK3d**

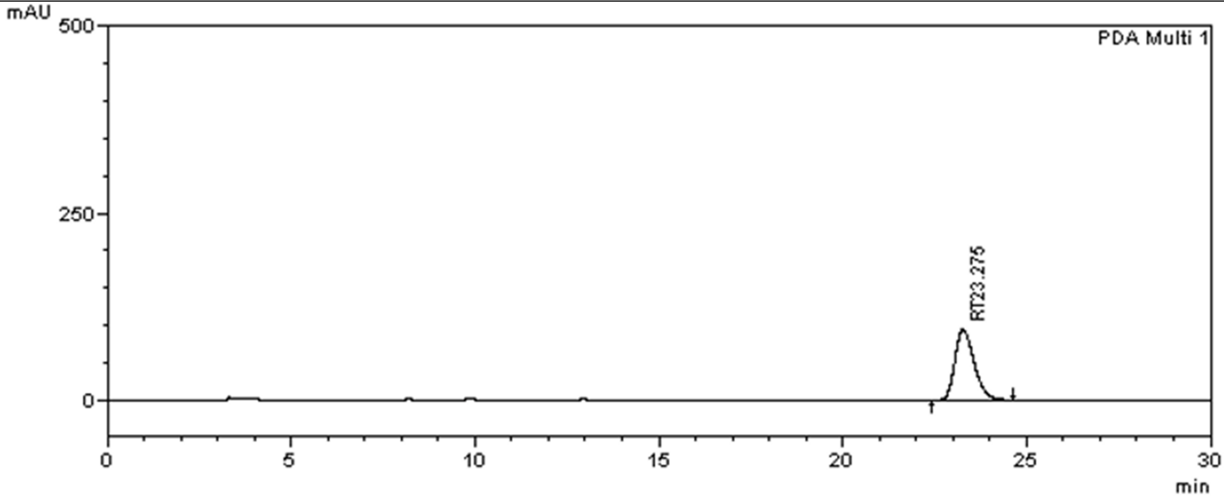


1 PDA Multi 1

<Results>

PDA Ch1 260nm 4nm

| Peak# | Ret. Time | Area | Height | Area % | Height % | Resolution |
|---|---|---|---|---|---|---|
| 1 | 23.275 | 3434602 | 92696 | 100.000 | 100.000 | 0.000 |
| Total | | 3434602 | 92696 | 100.000 | 100.000 | |

**Figure S12:** Purity chromatogram of the **VK3e**

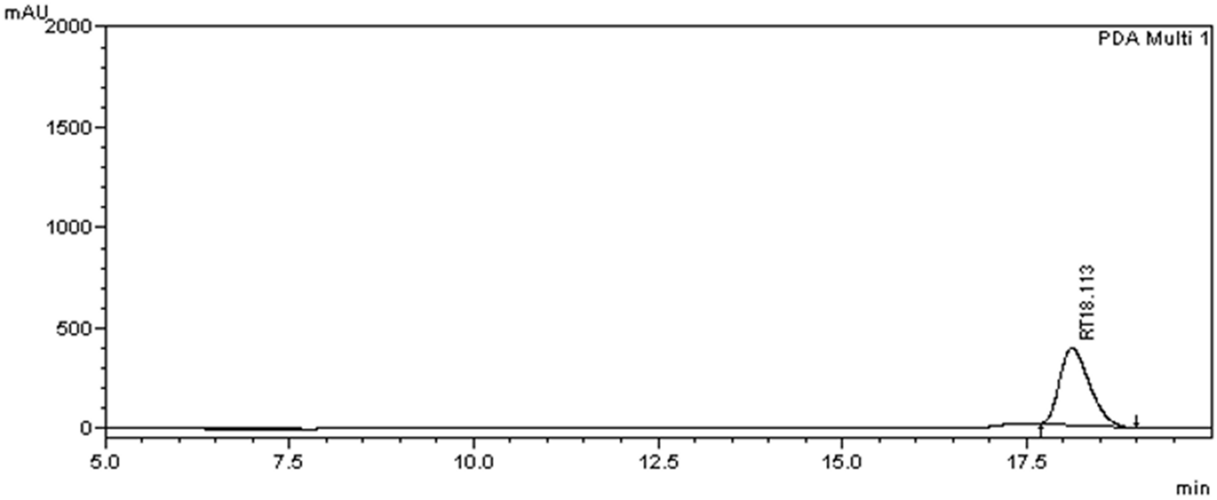


1 PDA Multi 1

<Results>

PDA Ch1 260nm 4nm

| Peak# | Ret. Time | Area | Height | Area % | Height % | Resolution |
|---|---|---|---|---|---|---|
| 1 | 18.113 | 10821659 | 382784 | 100.000 | 100.000 | 0.000 |
| Total | | 10821659 | 382784 | 100.000 | 100.000 | |

**Figure S13:** Purity chromatogram of the **VK3f**

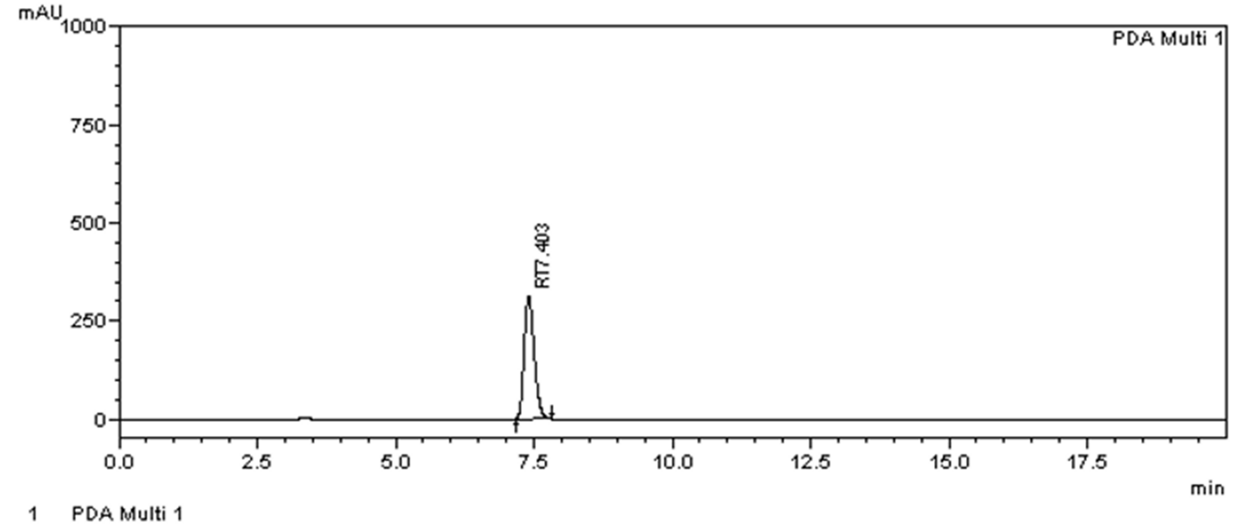


**<Results>**

PDA Ch1 260nm 4nm

| Peak# | Ret. Time | Area | Height | Area % | Height % | Resolution |
|---|---|---|---|---|---|---|
| 1 | 7.403 | 3992237 | 313539 | 100.000 | 100.000 | 0.000 |
| Total | | 3992237 | 313539 | 100.000 | 100.000 | |

**Figure S14:** Purity chromatogram of the **VK3g**

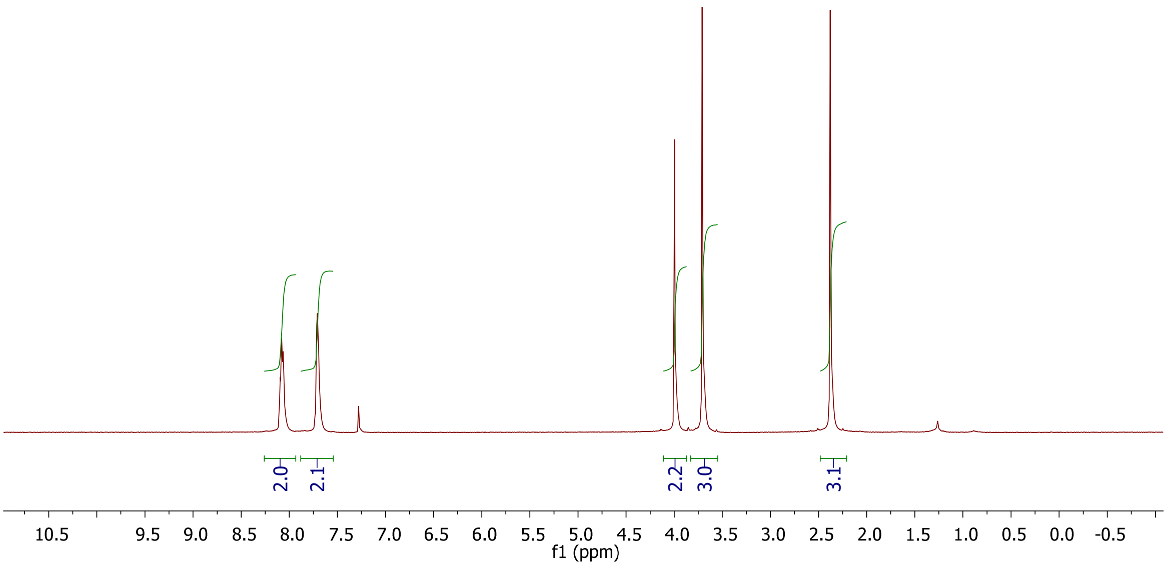


**Figure S15:** $^{1}$H NMR (500 MHz) spectrum of the **VK3a** in $CDCl_3$-*d1*

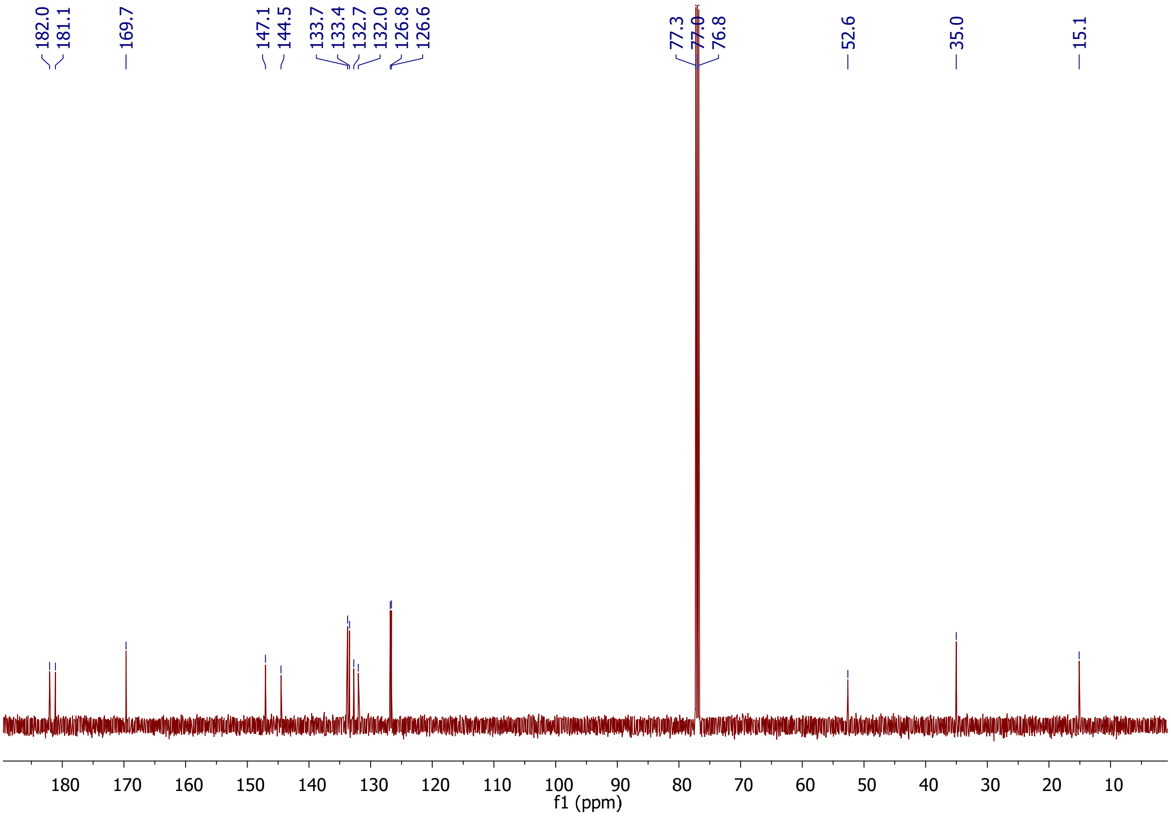


**Figure S16:** $^{13}$C NMR (125 MHz) spectrum of the **VK3a** in $CDCl_3$-*d1*

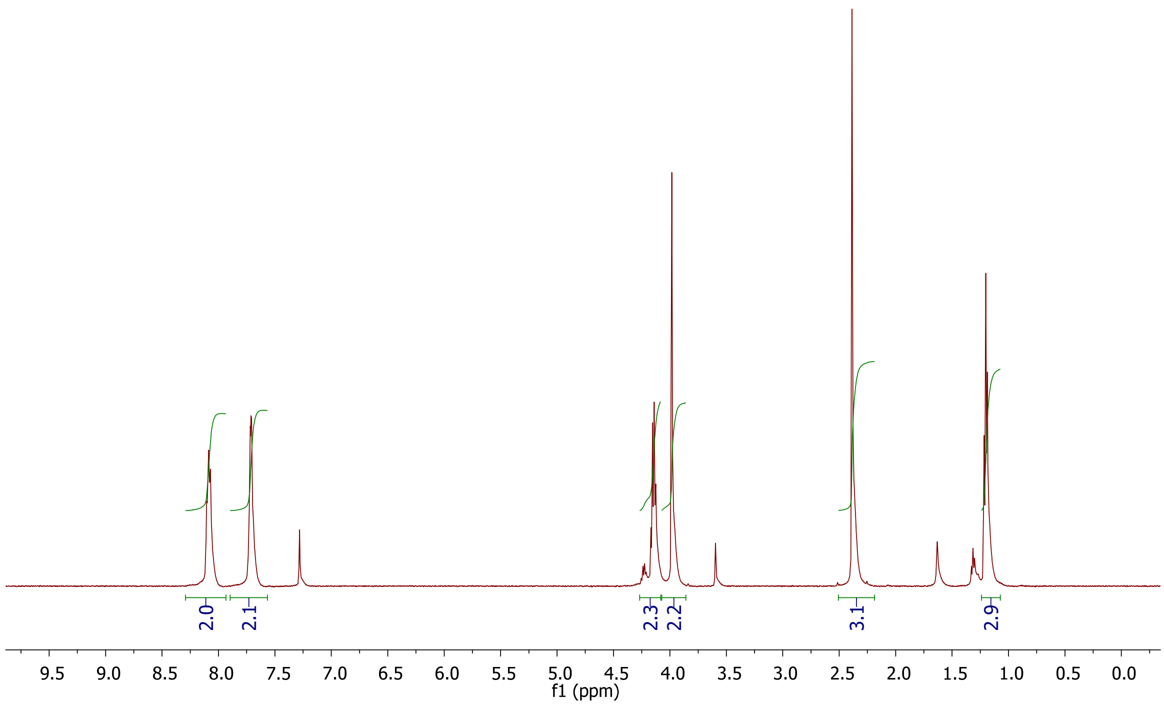


**Figure S17:** $^{1}$H NMR (500 MHz) spectrum of the **VK3b** in $CDCl_3$-*d1*

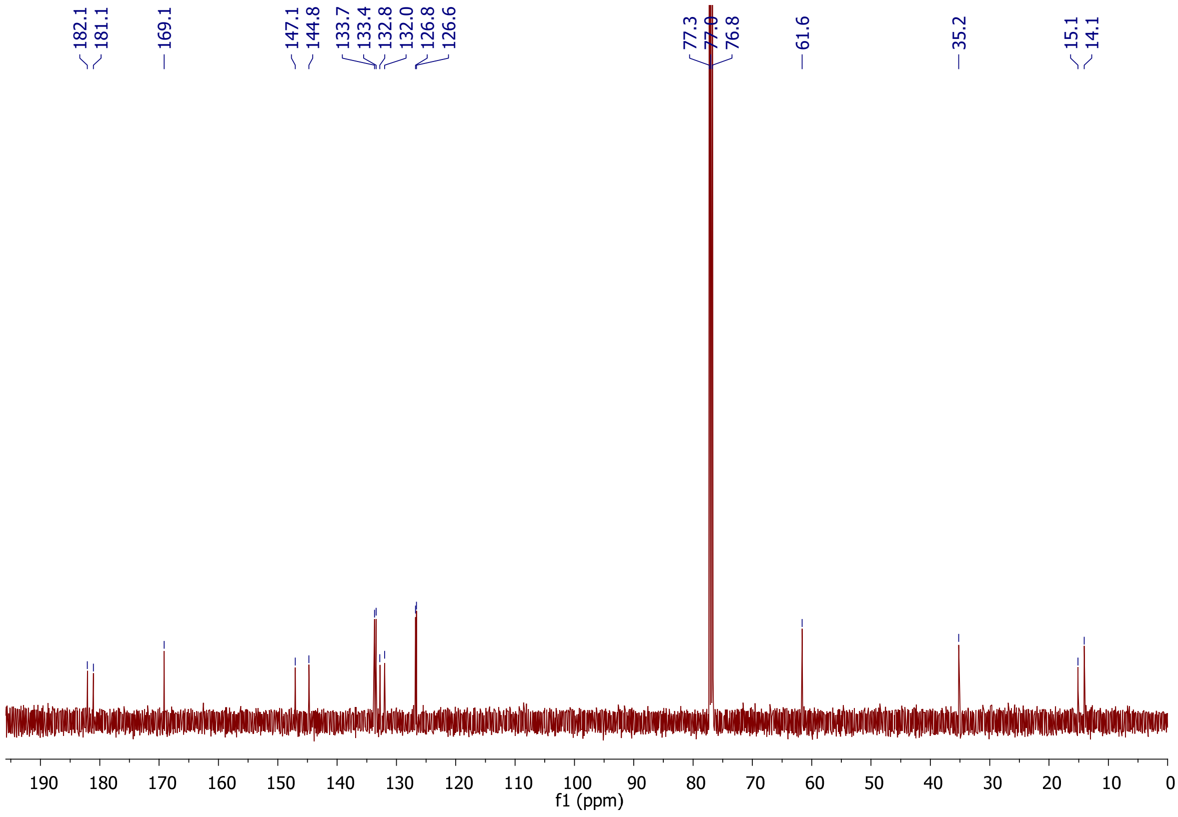


**Figure S18:** $^{13}$C NMR (125 MHz) spectrum of the **VK3b** in $CDCl_3$-*d1*

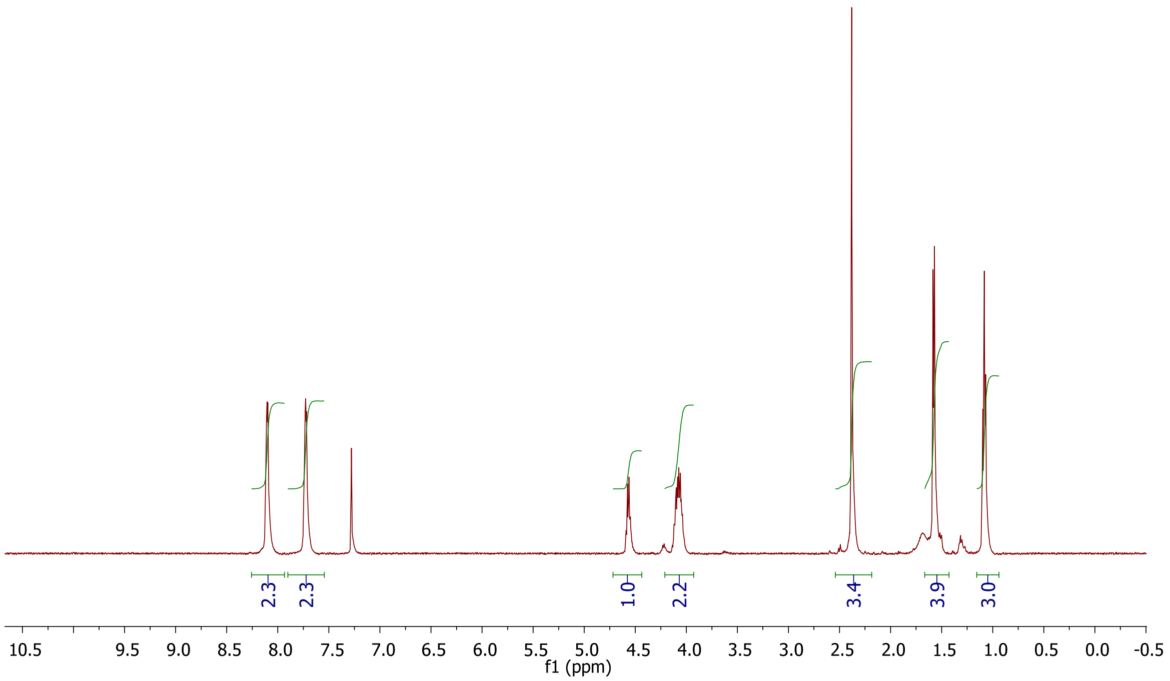


**Figure S19:** $^{1}$H NMR (500 MHz) spectrum of the **VK3c** in $CDCl_3$-*d1*

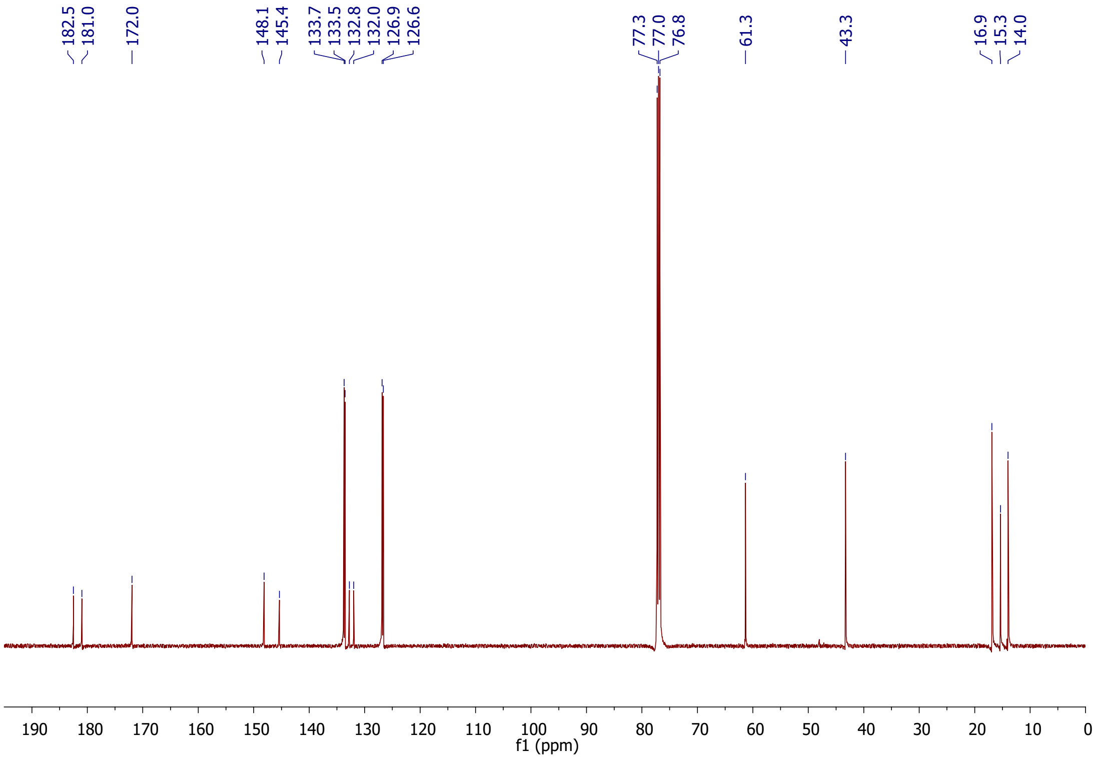


**Figure S20:** $^{13}$C NMR (125 MHz) spectrum of the **VK3c** in $CDCl_3$-*d1*

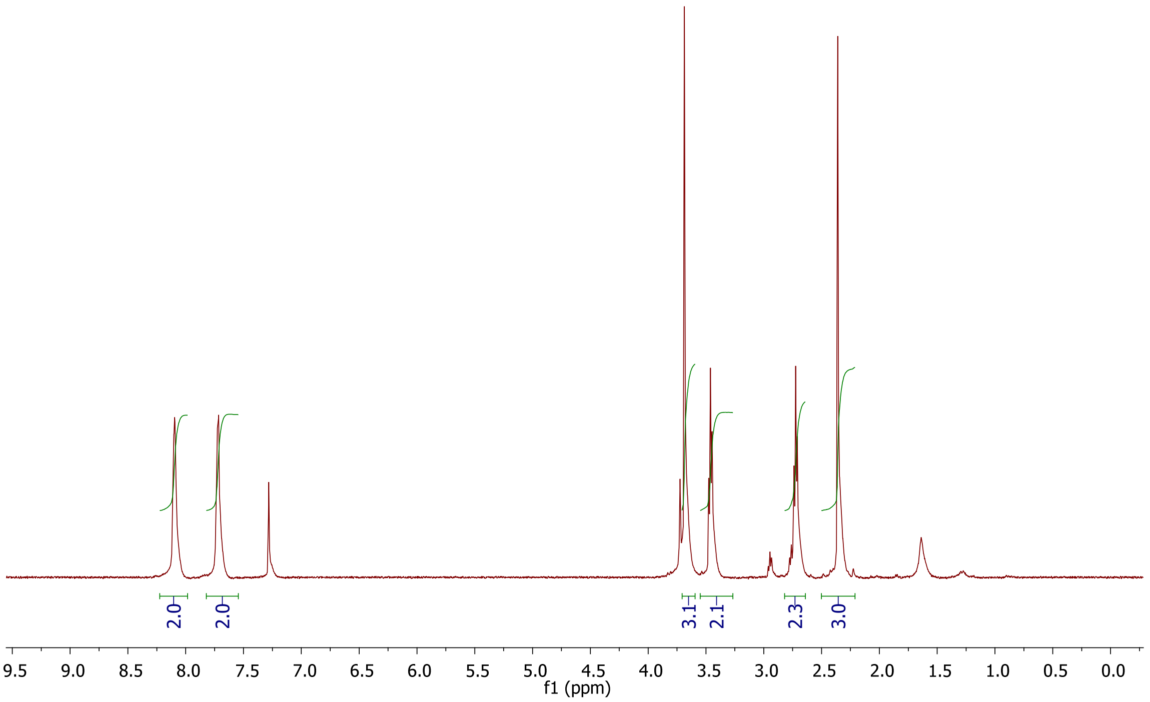


**Figure S21:** $^{1}$H NMR (500 MHz) spectrum of the **VK3d** in $CDCl_3$-*d1*

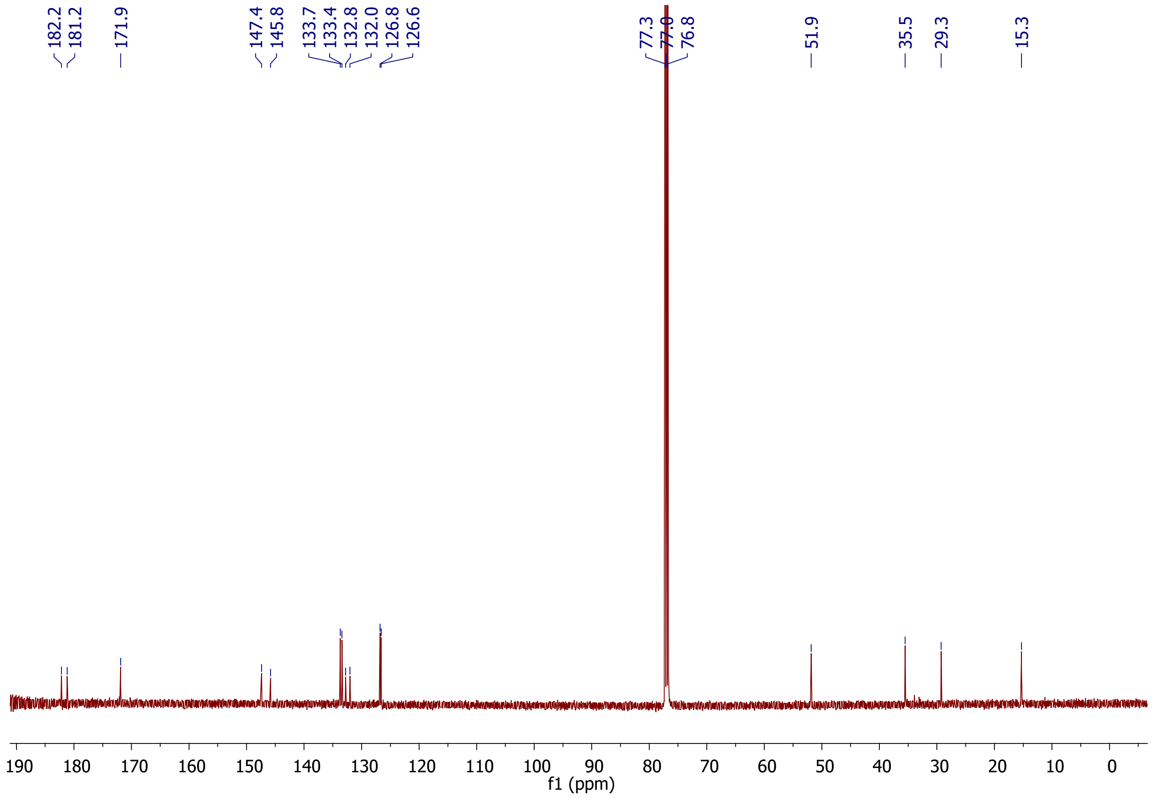


**Figure S22:** $^{13}$C NMR (125 MHz) spectrum of the **VK3d** in $CDCl_3$-*d1*

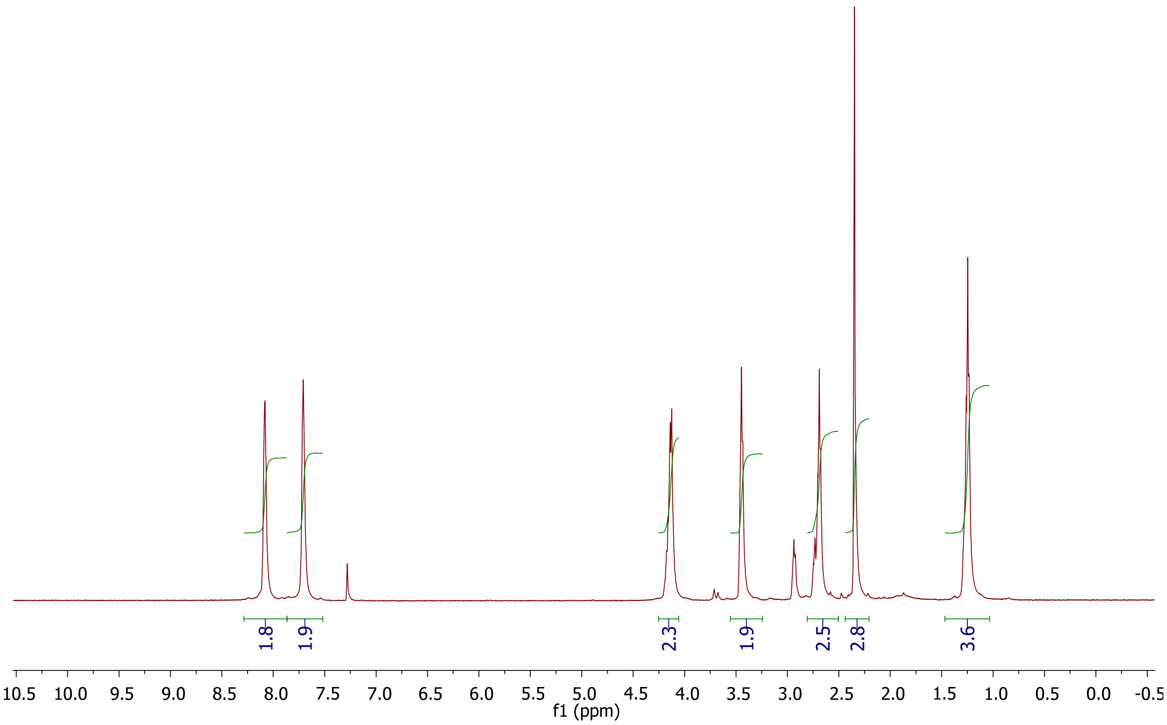


**Figure S23:** $^{1}$H NMR (500 MHz) spectrum of the **VK3e** in $CDCl_3$-*d1*

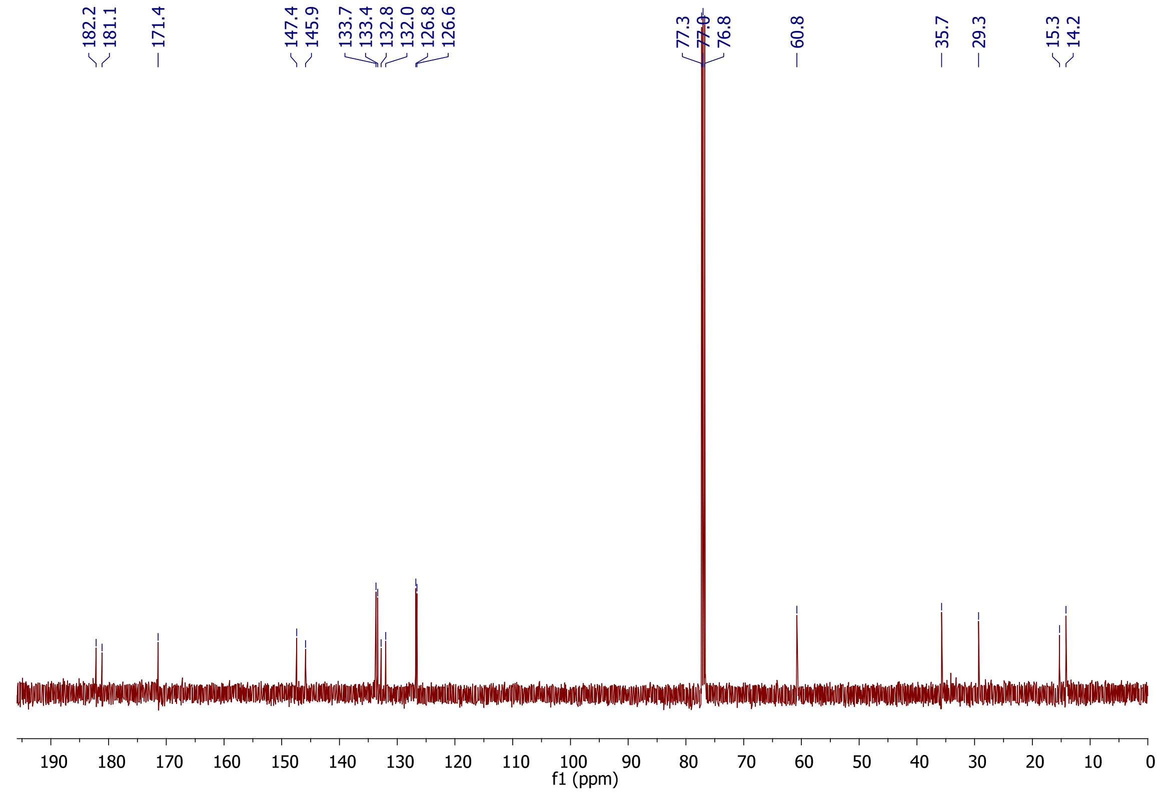


**Figure S24:** $^{13}$C NMR (125 MHz) spectrum of the **VK3e** in $CDCl_3$-*d1*

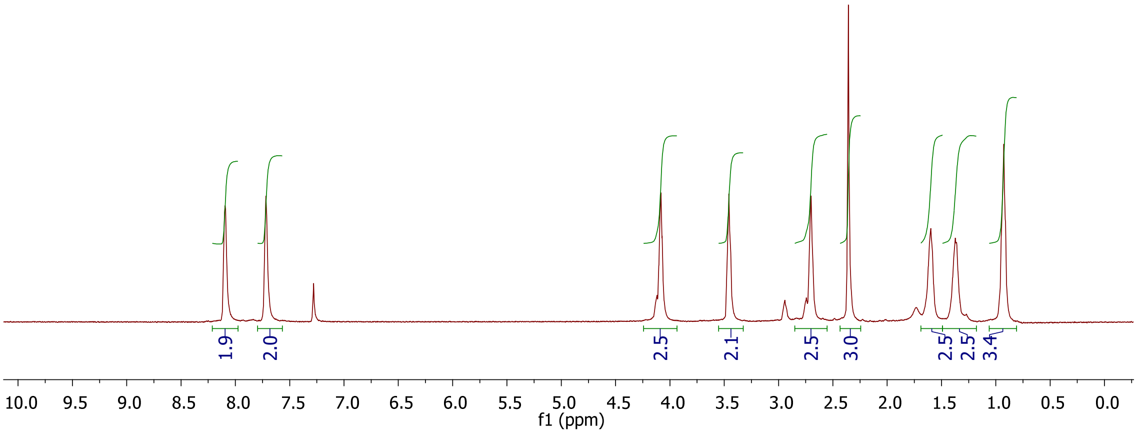


**Figure S25:** $^{1}$H NMR (500 MHz) spectrum of the **VK3f** in $CDCl_3$-*d1*

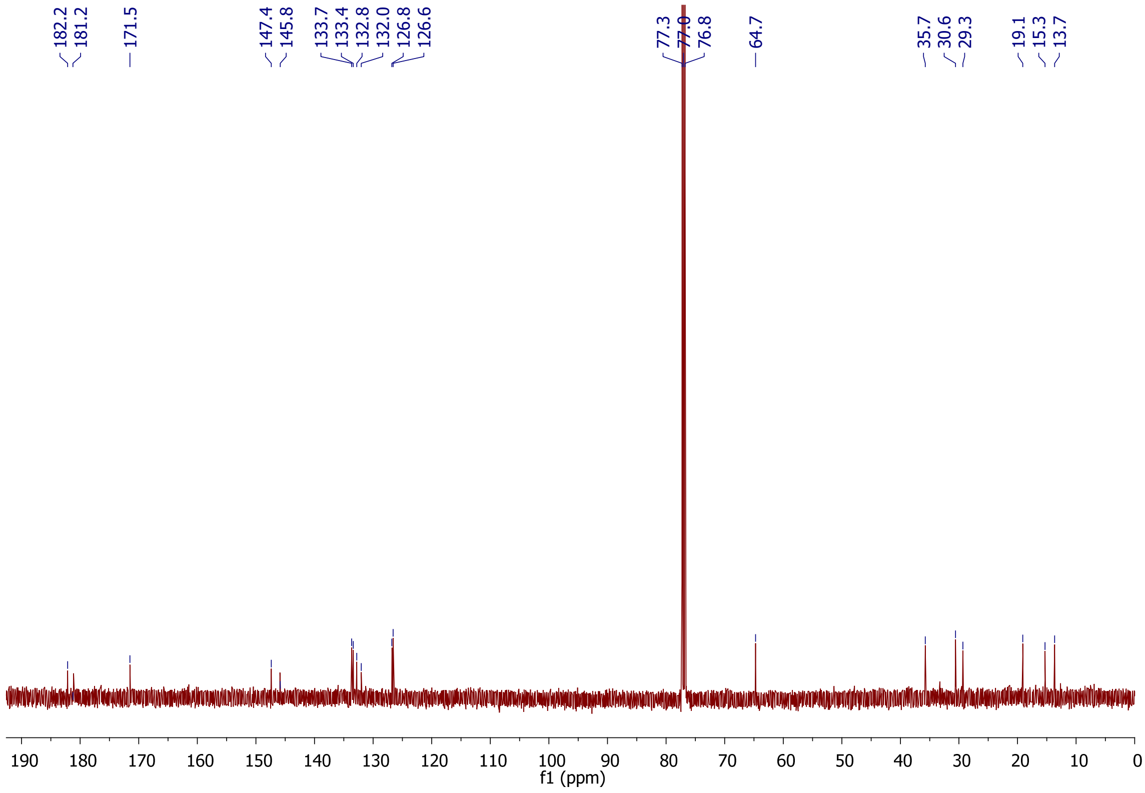


**Figure S26:** $^{13}$C NMR (125 MHz) spectrum of the **VK3f** in $CDCl_3$-*d1*

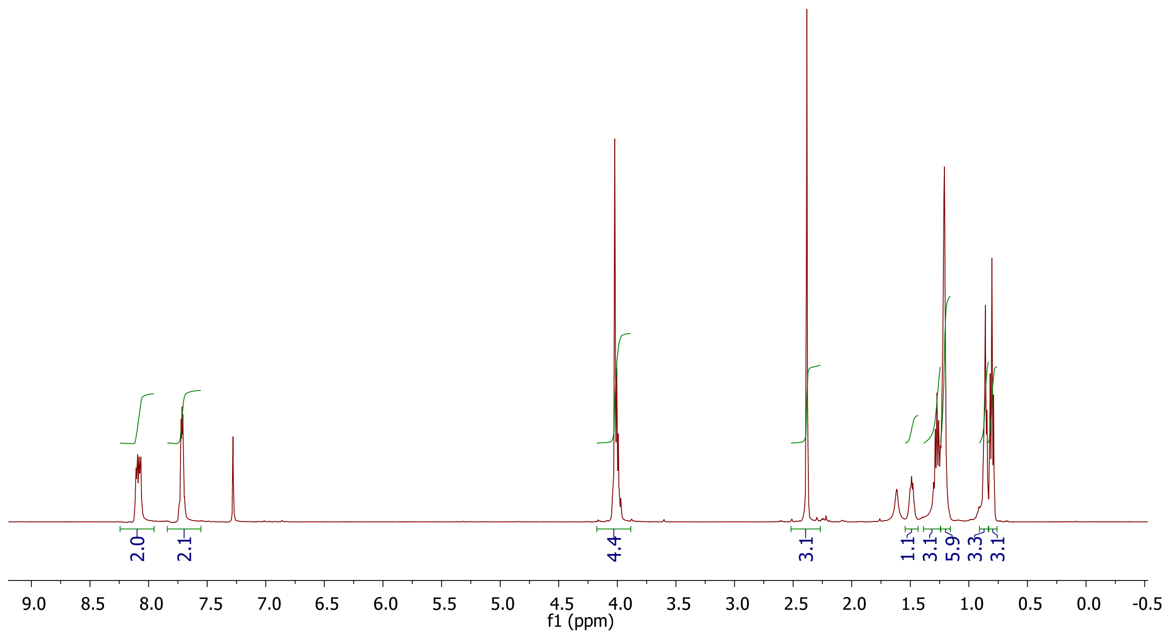


**Figure S27:** $^{1}$H NMR (500 MHz) spectrum of the **VK3g** in $CDCl_3$-*d1*

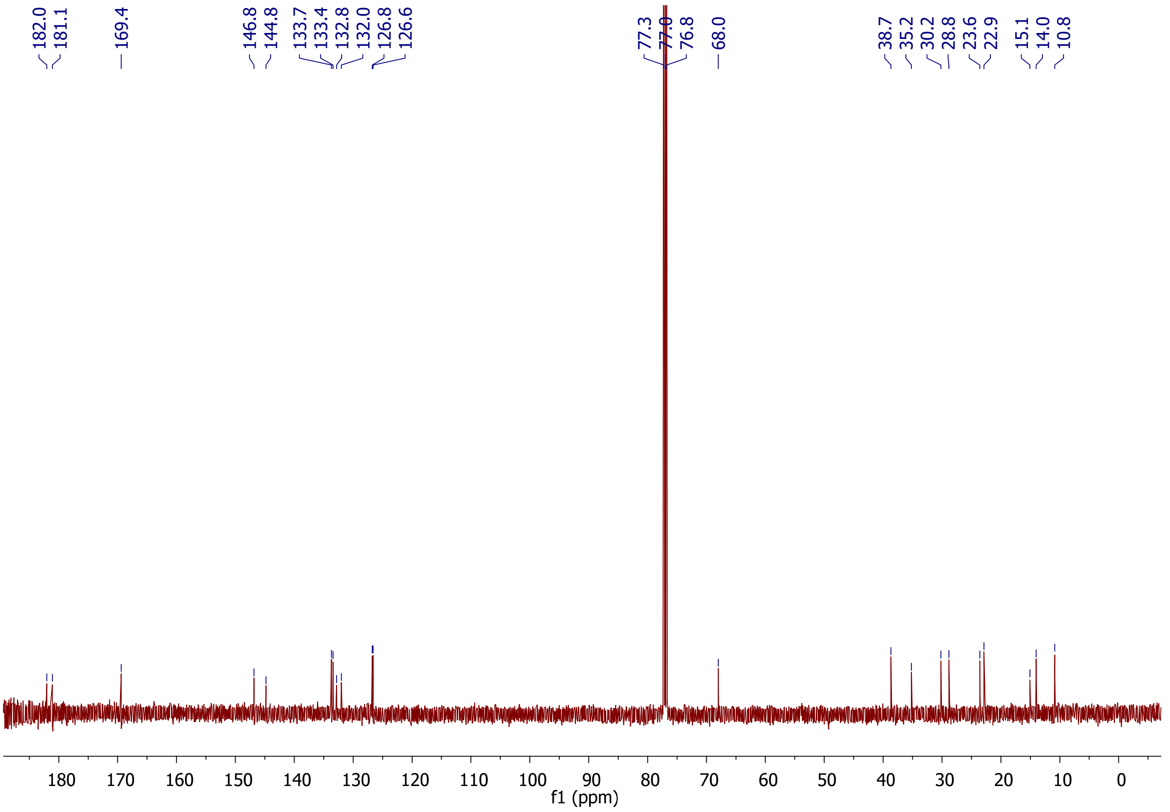


**Figure S28:** $^{13}$C NMR (125 MHz) spectrum of the **VK3g** in $CDCl_3$-*d1*

Crystallographic data of **VK3a**

| | |
|---|---|
| C14H12O4S<br>Mr = 276.30<br>Monoclinic, P21/n<br>a = 17.1194 (16) Å<br>b = 3.9184 (4) Å<br>c = 19.3277 (18) Å<br>β = 101.432 (1)°<br>V = 1270.8 (2) Å3<br>Z = 4<br>F(000) = 576<br>Dx = 1.444 Mg m−3<br>Mo Kα radiation, λ = 0.71073 Å<br>μ = 0.26 mm−1<br>T = 273 K<br>0.36 × 0.14 × 0.09 mm<br><br>Data collection<br>Bruker APEX II QUAZAR three-circle diffractometer<br>Radiation source: microfocus sealed tube, ImuS<br>Multilayer QUAZAR mirrors monochromator<br>φ and ω scans<br>15493 measured reflections<br>2896 independent reflections<br><br>2199 reflections with I > 2σ(I)<br>Rint = 0.056<br>θmax = 27.5°, θmin = 1.8°<br>h = −22→22<br>k = −5→5<br>l = −25→25 | Refinement<br>Refinement on F2<br>Least-squares matrix: full<br>R[F2 > 2σ(F2)] = 0.042<br>wR(F2) = 0.116<br>S = 1.02<br>2896 reflections<br>174 parameters<br>0 restraints<br>Hydrogen site location: inferred from neighbouring<br>sites<br>H-atom parameters constrained<br>w = 1/[σ2(Fo<br>2) + (0.0522P)2 + 0.4264P]<br>where P = (Fo<br>2 + 2Fc<br>2)/3<br>(Δ/σ)max < 0.001<br>Δρmax = 0.28 e Å−3<br>Δρmin = −0.23 e Å−3 |

**Table S1.** Bond lengths (Å) for **VK3a**

| | | | |
|---|---|---|---|
| S1-C4 | 1.7583(18) | S1-C3 | 1.8145(18) |
| O2-C2 | 1.325(2) | O2-C1 | 1.449(2) |
| O3-C14 | 1.218(2) | O1-C2 | 1.195(2) |
| O4-C7 | 1.216(2) | C4-C5 | 1.351(2) |
| C4-C14 | 1.495(2) | C13-C12 | 1.390(2) |
| C13-C8 | 1.398(2) | C13-C14 | 1.474(2) |
| C5-C7 | 1.480(2) | C5-C6 | 1.500(2) |
| C2-C3 | 1.500(3) | C8-C9 | 1.387(3) |
| C8-C7 | 1.484(2) | C3-H3A | 0.97 |
| C3-H3B | 0.97 | C12-C11 | 1.375(3) |
| C12-H12 | 0.93 | C6-H6A | 0.96 |
| C6-H6B | 0.96 | C6-H6C | 0.96 |
| C9-C10 | 1.378(3) | C9-H9 | 0.93 |
| C10-C11 | 1.377(3) | C10-H10 | 0.93 |
| C11-H11 | 0.93 | C1-H1A | 0.96 |

| | | | |
|---|---|---|---|
| C1-H1B | 0.96 | C1-H1C | 0.96 |

**Table S2.** Bond angles (°) for **VK3a**

| | | | |
|---|---|---|---|
| C4-S1-C3 | 102.70(8) | C2-O2-C1 | 116.90(16) |
| C5-C4-C14 | 121.34(16) | C5-C4-S1 | 119.68(13) |
| C14-C4-S1 | 118.61(12) | C12-C13-C8 | 119.37(17) |
| C12-C13-C14 | 120.21(16) | C8-C13-C14 | 120.41(15) |
| O3-C14-C13 | 121.74(16) | O3-C14-C4 | 120.22(16) |
| C13-C14-C4 | 118.00(15) | C4-C5-C7 | 120.61(15) |
| C4-C5-C6 | 123.16(17) | C7-C5-C6 | 116.23(15) |
| O1-C2-O2 | 124.19(18) | O1-C2-C3 | 122.32(18) |
| O2-C2-C3 | 113.48(16) | C9-C8-C13 | 119.95(16) |
| C9-C8-C7 | 120.16(16) | C13-C8-C7 | 119.89(16) |
| C2-C3-S1 | 112.18(12) | C2-C3-H3A | 109.2 |
| S1-C3-H3A | 109.2 | C2-C3-H3B | 109.2 |
| S1-C3-H3B | 109.2 | H3A-C3-H3B | 107.9 |
| O4-C7-C5 | 119.93(17) | O4-C7-C8 | 121.02(17) |
| C5-C7-C8 | 119.02(15) | C11-C12-C13 | 119.99(18) |
| C11-C12-H12 | 120.0 | C13-C12-H12 | 120.0 |
| C5-C6-H6A | 109.5 | C5-C6-H6B | 109.5 |
| H6A-C6-H6B | 109.5 | C5-C6-H6C | 109.5 |
| H6A-C6-H6C | 109.5 | H6B-C6-H6C | 109.5 |
| C10-C9-C8 | 119.83(18) | C10-C9-H9 | 120.1 |
| C8-C9-H9 | 120.1 | C11-C10-C9 | 120.33(19) |
| C11-C10-H10 | 119.8 | C9-C10-H10 | 119.8 |
| C12-C11-C10 | 120.53(19) | C12-C11-H11 | 119.7 |
| C10-C11-H11 | 119.7 | O2-C1-H1A | 109.5 |
| O2-C1-H1B | 109.5 | H1A-C1-H1B | 109.5 |
| O2-C1-H1C | 109.5 | H1A-C1-H1C | 109.5 |
| H1B-C1-H1C | 109.5 | | |

**Table S3.** Torsion angles (°) for **VK3a**

| | | | |
|---|---|---|---|
| C3-S1-C4-C5 | 137.42(16) | C3-S1-C4-C14 | -49.49(16) |
| C12-C13-C14-O3 | 9.6(3) | C8-C13-C14-O3 | -169.01(19) |
| C12-C13-C14-C4 | -172.84(18) | C8-C13-C14-C4 | 8.6(3) |
| C5-C4-C14-O3 | 167.60(19) | S1-C4-C14-O3 | -5.4(3) |
| C5-C4-C14-C13 | -10.0(3) | S1-C4-C14-C13 | 176.99(13) |
| C14-C4-C5-C7 | 4.8(3) | S1-C4-C5-C7 | 177.68(14) |
| C14-C4-C5-C6 | -176.20(18) | S1-C4-C5-C6 | -3.3(3) |
| C1-O2-C2-O1 | 0.2(3) | C1-O2-C2-C3 | -178.98(18) |
| C12-C13-C8-C9 | -0.3(3) | C14-C13-C8-C9 | 178.23(19) |
| C12-C13-C8-C7 | 179.24(19) | C14-C13-C8-C7 | -2.2(3) |
| O1-C2-C3-S1 | 164.08(18) | O2-C2-C3-S1 | -16.7(2) |

| | | | |
|---|---|---|---|
| C4-S1-C3-C2 | -151.94(14) | C4-C5-C7-O4 | 179.9(2) |
| C6-C5-C7-O4 | 0.8(3) | C4-C5-C7-C8 | 1.9(3) |
| C6-C5-C7-C8 | -177.17(18) | C9-C8-C7-O4 | -1.6(3) |
| C13-C8-C7-O4 | 178.8(2) | C9-C8-C7-C5 | 176.34(19) |
| C13-C8-C7-C5 | -3.2(3) | C8-C13-C12-C11 | 0.0(3) |
| C14-C13-C12-C11 | -178.62(19) | C13-C8-C9-C10 | 0.2(3) |
| C7-C8-C9-C10 | -179.4(2) | C8-C9-C10-C11 | 0.3(4) |
| C13-C12-C11-C10 | 0.6(4) | C9-C10-C11-C12 | -0.7(4) |
